# Bibliometric analysis and visualization of antidepressant-gut microbiota research: From preclinical exploration to clinical translation (2000–2026)

**DOI:** 10.1097/MD.0000000000051384

**Published:** 2026-10-30

**Authors:** Siliang Zeng, Wen Zheng, Dongfeng Wan, Huili Chen

**Affiliations:** a School of Health, Shanghai Normal University Tianhua College, Shanghai, China; b Translational Research Institute of Brain and Brain-Like Intelligence, Shanghai Fourth People’s Hospital Affiliated to Tongji University School of Medicine, Shanghai, China.

**Keywords:** antidepressant, bibliometric, CiteSpace, gut microbiota, hotspots, visualization, VOSviewer

## Abstract

**Background:**

Research on the interactions between antidepressants and the gut microbiota has expanded rapidly in recent years, with increasing attention to the microbiota-gut-brain axis and its potential relevance to antidepressant treatment. However, the overall development, knowledge structure, thematic evolution, and translational trajectory of this field remain insufficiently characterized. This study aimed to systematically map the research landscape of antidepressant-gut microbiota interactions and to identify major research hotspots, evolving themes, collaborative patterns, and emerging directions toward clinical translation.

**Methods:**

Publications from 2000 to 2026 were retrieved from the Web of Science Core Collection and Scopus. Bibliometric analyses were performed using R, VOSviewer, SCImago Graphica, and CiteSpace to evaluate publication trends, geographical and author collaboration, keyword co-occurrence and clustering, thematic evolution, co-citation patterns, and citation bursts. Relevant clinical studies were additionally identified through PubMed to complement the assessment of translational development.

**Results:**

A total of 1961 publications were included, with research output increasing markedly after 2017. China contributed the largest number of publications, whereas the United States demonstrated relatively extensive international collaboration. Keyword and clustering analyses identified depression, gut microbiota, probiotics, the gut-brain axis, inflammation, and antidepressant-related mechanisms as major research domains. Thematic evolution showed a progression from early research centered on gastrointestinal disorders toward increasingly diverse investigations involving depression, stress, probiotics, and microbial mechanisms, followed by recent convergence around the gut-brain axis, antidepressants, and neuroinflammation. Highly cited and citation-burst references further reflected the growing influence of mechanistic and microbiota-targeted research. Clinical studies have increasingly investigated microbiota-related interventions and mechanistic biomarkers alongside antidepressant treatment, although the available evidence remains heterogeneous.

**Conclusion:**

Antidepressant-gut microbiota research has undergone substantial expansion and thematic transformation, with increasing emphasis on mechanistic pathways and clinical relevance. The field is progressing from exploratory associations toward mechanism-informed and translational research, although further standardized clinical investigation is needed to establish the therapeutic and predictive relevance of the gut microbiota in antidepressant treatment.

## 1. Introduction

Depression is a prevalent mental disorder and a major contributor to the global public health burden. In 2015, an estimated 264 million people worldwide, representing approximately 3.6% of the global population, were living with depression.^[1]^ More recent data from the Global Burden of Disease study further highlight this growing burden, showing that the age-standardized disability-adjusted life-year rate for depressive disorders increased by 26.3% between 2010 and 2023, placing depression among the non-communicable diseases with the most pronounced increases in disease burden.^[2]^ Serotonergic antidepressants, particularly selective serotonin reuptake inhibitors and serotonin-norepinephrine reuptake inhibitors, remain among the primary pharmacological treatments for major depressive disorder.^[3]^ However, their clinical use is limited by several challenges, including gastrointestinal and sexual adverse effects, delayed onset of therapeutic action, and considerable interindividual variability in treatment response.^[3,4]^ These limitations not only compromise treatment adherence and quality of life but also underscore the need for complementary strategies to optimize antidepressant treatment. In this context, the gut microbiota has attracted increasing attention as a potential modulator of antidepressant response.

Accumulating evidence suggests that interactions between the gut microbiome and therapeutic drugs are bidirectional. On 1 hand, gut microorganisms can influence drug disposition and bioavailability through processes such as microbial metabolism, chemical modification, and bioaccumulation.^[5,6]^ On the other hand, drug exposure can alter microbial composition, metabolic activity, and community ecology.^[7–10]^ For example, Zimmermann et al systematically assessed the capacity of 76 representative human gut bacterial strains to metabolize 271 orally administered drugs and found that many compounds underwent direct microbial chemical modification, supporting a broader role of the gut microbiome in shaping drug disposition and interindividual variability in drug response.^[7]^ More specifically, Klünemann et al further demonstrated that several representative gut bacterial strains could deplete the widely used antidepressant duloxetine from the culture medium through intracellular bioaccumulation rather than biotransformation. Duloxetine exposure also reshaped the composition of a defined microbial community through alterations in bacterial metabolism and metabolic cross-feeding.^[5]^

Research in this field has expanded rapidly across mechanistic, preclinical, and clinical domains in the past 2 decades, yet its evolving research landscape and clinical translation remain insufficiently characterized. Bibliometric analysis provides a quantitative approach to systematically characterize the development and structure of a research field by mapping scientific collaboration, research themes, and emerging trends.^[11]^ It can therefore offer a comprehensive perspective on the evolution of a field and help identify underexplored areas and future research directions.^[12–14]^ Accordingly, we conducted a bibliometric analysis of 1961 publications on antidepressants and the gut microbiota from the Web of Science and Scopus databases between 2000 and 2026, complemented by a systematic assessment of 13 clinical trials identified in PubMed. By integrating collaboration networks, keyword and thematic analyses, and clinical evidence, this study aimed to characterize the development and emerging directions of the field and to assess the progression of antidepressant-gut microbiota research from preclinical exploration toward clinical investigation.

## 2. Methods

### 2.1. Study design and database

The year 2000 was selected as the starting point to capture the contemporary development of antidepressant-gut microbiota research over a sufficiently long observation period. Accordingly, bibliometric data were retrieved from 2 major databases, the Web of Science Core Collection (WoSCC); (Clarivate, Philadelphia) and Scopus (Elsevier, Amsterdam, the Netherlands), covering publications from January 1, 2000, to September 1, 2026. To minimize potential discrepancies caused by continuous database updates, all literature searches and data downloads were completed on September 1, 2026. The search strategy was constructed around 4 groups of terms covering depression (T1), antidepressants (T2), specific antidepressant agents (T3), and the gut microbiota (T4). Detailed database-specific search strategies for WoSCC and Scopus are provided in Tables 1–2, respectively. Only articles and review articles published in English were included. To further assess the clinical translation of antidepressant-gut microbiota research, PubMed was systematically searched for relevant clinical trials and randomized controlled trials involving antidepressants and the gut microbiota. For each eligible study, the following information was extracted: publication title, first author, journal, trial registration information, study design, study population, antidepressant treatment, microbiota-related intervention, follow-up duration, and key findings. The detailed PubMed search strategy is provided in Table 3, and the screening and selection processes for both the bibliometric literature and clinical trials are presented in Figure 1.

**Table 1:** Search strategy for Web of Science Core Collection database.

Search strategy for Web of Science Core Collection		
#1	TS = (depress* OR dysthymi* OR “mood disorder*” OR “affective disorder*” OR “major depressive disorder*” OR MDD OR “depressive symptom*”)	1102,437
#2	TS = (antidepress* OR anti-depress* OR MAOI* OR “monoamine oxidase inhibit*” OR RIMA* OR “reversible inhibitor* of monoamine oxidase A” OR (serotonin OR norepinephrine OR noradrenaline OR neurotransmitter* OR dopamin*) AND (uptake OR reuptake OR re-uptake) OR SSRI* OR “selective serotonin reuptake inhibit*” OR SNRI* OR “serotonin norepinephrine reuptake inhibit*” OR “serotonin-norepinephrine reuptake inhibit*” OR NaSSA* OR “noradrenergic and specific serotonergic antidepressant*” OR NARI* OR “noradrenaline reuptake inhibit*” OR “norepinephrine reuptake inhibit*” OR SARI* OR “serotonin antagonist and reuptake inhibit*” OR NDRI* OR “norepinephrine dopamine reuptake inhibit*” OR “norepinephrine-dopamine reuptake inhibit*” OR TCA* OR tricyclic* OR tetracyclic* OR heterocyclic*)	371,465
#3	TS = (Agomelatine OR alaproclate OR amersergide OR amfebutamone OR bupropion OR amineptine OR amitriptyline OR amitriptylinoxide OR amoxapine OR atomoxetine OR befloxatone OR benactyzine OR binospirone OR brofaromine OR buspirone OR butriptyline OR caroxazone OR clomipramin* OR chlorpoxiten OR cianopramine OR cilobamine OR cimoxatone OR citalopram* OR clorgyline OR clorimipramine OR clovoxamine OR demexiptiline OR deprenyl OR desipramine OR pertrofrane OR desvenlafaxine OR dibenzepin OR dibenzipin OR diclofensine OR dimetacrin* OR dosulepin OR dothiepin OR doxepin OR duloxetine OR edivoxetine OR escitalopram OR etoperidone OR femoxetine OR fluotracen OR fluoxetine OR fluvoxamine OR imipramine OR iprindole OR iproniazid* OR ipsapirone OR isocarboxazid* OR ketamine OR esketamine OR lanicemine OR levomilnacipran OR lofepramine OR lopramine OR maprotiline OR melitracen OR metapramine OR mianserin OR milnacipran OR minaprine OR mirtazapine OR moclobemide OR nefazodone OR nialamide OR nitroxazepine OR nomifensine OR norfenfluramine OR nortriptylin* OR noxiptilin* OR opipramol OR oxaflozane OR paroxetine OR phenelzine OR pheniprazine OR pipofezine OR pirlindole OR propizepine OR protriptyline OR quinupramine OR reboxetine OR rolipram OR scopolamine OR selegiline OR sertraline OR setiptiline OR tandospirone OR teciptiline OR thozalinone OR tianeptine OR toloxatone OR tranylcypromin* OR trazodone OR trimipramine OR venlafaxine OR vilazodone OR viloxazine OR viqualine OR vortioxetine OR zalospirone OR “traditional chinese medicine*” OR TCM*)	241,800
#4	#2 OR #3	552,045
#5	TS = (gut OR intestinal OR intestine OR gastrointestinal OR enteric) AND (microb* OR microbiom* OR microbiot* OR bacteria* OR flora OR microflora OR ecosystem OR dysbiosis)	300,369
#6	#1 AND #4 AND #5	1259

**Table 2:** Search strategy for Scopus database.

Search strategy for Scopus		
#1	TITLE-ABS-KEY = (depress* OR dysthymi* OR “mood disorder*” OR “affective disorder*” OR “major depressive disorder*” OR MDD OR “depressive symptom*”)	1348,050
#2	TITLE-ABS-KEY = (antidepress* OR anti-depress* OR MAOI* OR “monoamine oxidase inhibit*” OR RIMA* OR “reversible inhibitor* of monoamine oxidase A” OR (serotonin OR norepinephrine OR noradrenaline OR neurotransmitter* OR dopamin*) AND (uptake OR reuptake OR re-uptake) OR SSRI* OR “selective serotonin reuptake inhibit*” OR SNRI* OR “serotonin norepinephrine reuptake inhibit*” OR “serotonin-norepinephrine reuptake inhibit*” OR NaSSA* OR “noradrenergic and specific serotonergic antidepressant*” OR NARI* OR “noradrenaline reuptake inhibit*” OR “norepinephrine reuptake inhibit*” OR SARI* OR “serotonin antagonist and reuptake inhibit*” OR NDRI* OR “norepinephrine dopamine reuptake inhibit*” OR “norepinephrine-dopamine reuptake inhibit*” OR TCA* OR tricyclic* OR tetracyclic* OR heterocyclic*	571,433
#3	TITLE-ABS-KEY = (Agomelatine OR alaproclate OR amersergide OR amfebutamone OR bupropion OR amineptine OR amitriptyline OR amitriptylinoxide OR amoxapine OR atomoxetine OR befloxatone OR benactyzine OR binospirone OR brofaromine OR buspirone OR butriptyline OR caroxazone OR clomipramin* OR chlorpoxiten OR cianopramine OR cilobamine OR cimoxatone OR citalopram* OR clorgyline OR clorimipramine OR clovoxamine OR demexiptiline OR deprenyl OR desipramine OR pertrofrane OR desvenlafaxine OR dibenzepin OR dibenzipin OR diclofensine OR dimetacrin* OR dosulepin OR dothiepin OR doxepin OR duloxetine OR edivoxetine OR escitalopram OR etoperidone OR femoxetine OR fluotracen OR fluoxetine OR fluvoxamine OR imipramine OR iprindole OR iproniazid* OR ipsapirone OR isocarboxazid* OR ketamine OR esketamine OR lanicemine OR levomilnacipran OR lofepramine OR lopramine OR maprotiline OR melitracen OR metapramine OR mianserin OR milnacipran OR minaprine OR mirtazapine OR moclobemide OR nefazodone OR nialamide OR nitroxazepine OR nomifensine OR norfenfluramine OR nortriptylin* OR noxiptilin* OR opipramol OR oxaflozane OR paroxetine OR phenelzine OR pheniprazine OR pipofezine OR pirlindole OR propizepine OR protriptyline OR quinupramine OR reboxetine OR rolipram OR scopolamine OR selegiline OR sertraline OR setiptiline OR tandospirone OR teciptiline OR thozalinone OR tianeptine OR toloxatone OR tranylcypromin* OR trazodone OR trimipramine OR venlafaxine OR vilazodone OR viloxazine OR viqualine OR vortioxetine OR zalospirone OR “traditional chinese medicine*” OR TCM*)	467,761
#4	#2 OR #3	909,107
#5	TITLE-ABS-KEY=(gut OR intestinal OR intestine OR gastrointestinal OR enteric) AND (microb* OR microbiom* OR microbiot* OR bacteria* OR flora OR microflora OR ecosystem OR dysbiosis)	387,411
#6	#1 AND #4 AND #5	2009

**Table 3:** Search strategy for PubMed database.

Search strategy for PubMed		
#1	“Depressive Disorder”[MeSH Terms] OR “Depression”[MeSH Terms] OR “depress*”[Title/Abstract] OR “dysthymi*”[Title/Abstract] OR “mood disorder”[Title/Abstract] OR “mood disorders”[Title/Abstract] OR “affective disorder”[Title/Abstract] OR “affective disorders”[Title/Abstract] OR “major depressive disorder”[Title/Abstract] OR “major depressive disorders”[Title/Abstract] OR “MDD”[Title/Abstract] OR “depressive symptom”[Title/Abstract] OR “depressive symptoms”[Title/Abstract]	754,527
#2	“Antidepressive Agents”[MeSH Terms] OR “antidepress*”[Title/Abstract] OR “anti-depressant”[Title/Abstract] OR “anti-depressants”[Title/Abstract] OR “monoamine oxidase inhibitor”[Title/Abstract] OR “monoamine oxidase inhibitors”[Title/Abstract] OR “MAOI”[Title/Abstract] OR “MAOIs”[Title/Abstract] OR “selective serotonin reuptake inhibitor”[Title/Abstract] OR “selective serotonin reuptake inhibitors”[Title/Abstract] OR “SSRI”[Title/Abstract] OR “SSRIs”[Title/Abstract] OR “serotonin-norepinephrine reuptake inhibitor”[Title/Abstract] OR “serotonin-norepinephrine reuptake inhibitors”[Title/Abstract] OR “serotonin-norepinephrine reuptake inhibitor”[Title/Abstract] OR “serotonin-norepinephrine reuptake inhibitors”[Title/Abstract] OR “SNRI”[Title/Abstract] OR “SNRIs”[Title/Abstract] OR “tricyclic antidepressant”[Title/Abstract] OR “tricyclic antidepressants”[Title/Abstract] OR “tetracyclic antidepressant”[Title/Abstract] OR “tetracyclic antidepressants”[Title/Abstract] OR “agomelatine”[Title/Abstract] OR “bupropion”[Title/Abstract] OR “amitriptyline”[Title/Abstract] OR “clomipramine”[Title/Abstract] OR “citalopram”[Title/Abstract] OR “desipramine”[Title/Abstract] OR “desvenlafaxine”[Title/Abstract] OR “doxepin”[Title/Abstract] OR “duloxetine”[Title/Abstract] OR “escitalopram”[Title/Abstract] OR “fluoxetine”[Title/Abstract] OR “fluvoxamine”[Title/Abstract] OR “imipramine”[Title/Abstract] OR “ketamine”[Title/Abstract] OR “esketamine”[Title/Abstract] OR “levomilnacipran”[Title/Abstract] OR “maprotiline”[Title/Abstract] OR “mianserin”[Title/Abstract] OR “milnacipran”[Title/Abstract] OR “mirtazapine”[Title/Abstract] OR “moclobemide”[Title/Abstract] OR “nefazodone”[Title/Abstract] OR “nortriptyline”[Title/Abstract] OR “paroxetine”[Title/Abstract] OR “phenelzine”[Title/Abstract] OR “reboxetine”[Title/Abstract] OR “selegiline”[Title/Abstract] OR “sertraline”[Title/Abstract] OR “tianeptine”[Title/Abstract] OR “trazodone”[Title/Abstract] OR “tranylcypromine”[Title/Abstract] OR “trimipramine”[Title/Abstract] OR “venlafaxine”[Title/Abstract] OR “vilazodone”[Title/Abstract] OR “vortioxetine”[Title/Abstract]	190,405
#3	“Gastrointestinal Microbiome”[MeSH Terms] OR “Probiotics”[MeSH Terms] OR “Prebiotics”[MeSH Terms] OR “Fecal Microbiota Transplantation”[MeSH Terms] OR “gut microbiota”[Title/Abstract] OR “gut microbiome”[Title/Abstract] OR “intestinal microbiota”[Title/Abstract] OR “intestinal microbiome”[Title/Abstract] OR “gastrointestinal microbiota”[Title/Abstract] OR “Gastrointestinal Microbiome”[Title/Abstract] OR “gut bacteria”[Title/Abstract] OR “intestinal bacteria”[Title/Abstract] OR “gut flora”[Title/Abstract] OR “intestinal flora”[Title/Abstract] OR “dysbiosis”[Title/Abstract] OR “probiotic*”[Title/Abstract] OR “prebiotic*”[Title/Abstract] OR “psychobiotic*”[Title/Abstract] OR “synbiotic*”[Title/Abstract] OR “Fecal Microbiota Transplantation”[Title/Abstract] OR “fecal microbiota transplantation”[Title/Abstract] OR “fecal microbiota transplant”[Title/Abstract] OR “fecal microbiota transplant”[Title/Abstract] OR “FMT”[Title/Abstract]	200,926
#4	#1 AND #2 AND #3	903

**Figure 1.**
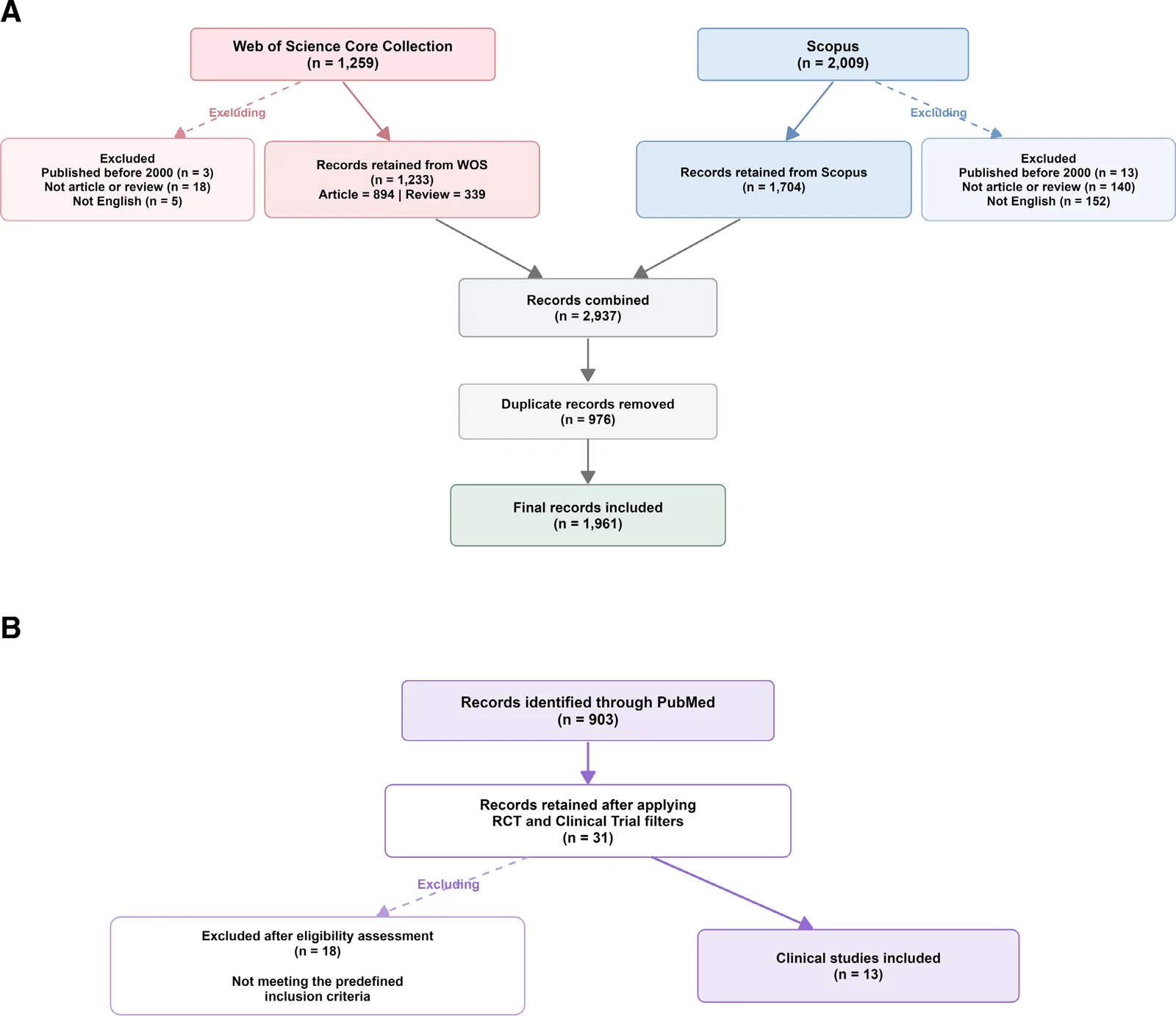
Study selection process. (A) Literature screening flowchart for bibliometric analysis. (B) Screening flowchart for clinical trials.

### 2.2. Statistical analysis

Data processing and bibliometric analyses were performed in R (version 4.5.2) using the bibliometrix and igraph packages. Records retrieved from WoSCC and Scopus were imported with bibliometrix and harmonized into a common bibliographic format. Duplicate records were identified sequentially using standardized DOIs and titles. DOIs were normalized by removing prefixes and whitespace and converting all characters to lowercase, while titles were normalized by converting them to lowercase, replacing punctuation with spaces, and standardizing whitespace. DOI-based deduplication was performed first, followed by title-based matching to identify additional duplicates. When duplicate records were identified across databases, the WoSCC record was preferentially retained; within the same database, the first occurrence was retained.

Following deduplication, the resulting dataset was analyzed to characterize publication output, temporal trends, and network-level bibliometric indicators. Annual publication output was calculated to characterize the temporal development of the field. Because the literature search was completed on September 1, 2026, the 2026 publication count represented a partial-year observation and was excluded from trend modeling. A third-order polynomial regression incorporating year, year^2^, and year^3^ terms was fitted to complete annual publication counts from 2000 to 2025, with 95% confidence intervals displayed around the fitted curve. The coefficient of determination (*R*^*2*^) was reported to assess model fit. The observed 2026 publication count was retained for descriptive presentation only and was interpreted as publications indexed up to the search date. Country-level scientific production was further classified into single-country publications and multiple-country publications (MCPs). single-country publications referred to publications in which all authors were affiliated with institutions from the same country, whereas MCPs represented publications involving authors from more than 1 country and were used to reflect international research collaboration.

Scientific collaboration and keyword co-occurrence structures were further explored using VOSviewer (version 1.6.20, Leiden University, Leiden, the Netherlands) and SCImago Graphica (version 1.0.55, SCImago Lab, Granada, Spain), with the latter used specifically for visualization of international collaboration patterns. VOSviewer analyses used full counting; authors with at least 5 publications and keywords with at least 5 occurrences were included. Association strength normalization was applied, with a clustering resolution of 1.00 and a minimum cluster size of 1. Before keyword-based analyses, the keyword list was manually reviewed to reduce terminological redundancy. Synonymous and conceptually equivalent terms were merged and represented by a single preferred term. For example, “major depression” was merged with “major depressive disorder,” “microbiota-gut-brain axis” was merged with “gut-brain axis,” and “animal experiment” was merged with “animal model.” The normalized keyword list was subsequently used for keyword co-occurrence, clustering, and thematic evolution analyses. In the resulting network maps, node size represents occurrence frequency, colors indicate cluster membership, and link thickness reflects the strength of connections between nodes. To characterize the intellectual and temporal structure of the field, CiteSpace (version 7.0.R1, Drexel University, Philadelphia) was used for co-citation analysis, citation burst detection, and temporal knowledge mapping. CiteSpace analyses covered 2000 to 2026 with 1 year per time slice; nodes were selected using the g-index (k = 17), no pruning procedures were applied, and cluster labels were generated using the log-likelihood ratio method. The modularity *Q* and weighted mean silhouette values were 0.4678 and 0.7065, respectively. Thematic evolution was additionally examined using the Biblioshiny interface within bibliometrix and visualized as a Sankey diagram. To better balance publication volumes across developmental stages, the study period was divided according to the temporal distribution of publications into 4 unequal intervals: 2000 to 2007, 2008 to 2014, 2015 to 2020, and 2021 to 2026, consistent with bibliometric methodological recommendations for maintaining comparable analytical capacity across periods.^[15]^

For the clinical translational analysis, 2 authors (SZ and WZ) independently screened the titles, abstracts, and full texts of potentially eligible studies. Studies were included if they were human clinical intervention studies involving participants with depressive disorders, incorporated an antidepressant treatment, and directly evaluated the relationship between antidepressant treatment and the gut microbiota through microbiota-related interventions, measurements, or outcomes. Studies involving anxiety disorders without depression, lacking an antidepressant treatment component, or not assessing gut microbiota-related factors were excluded. Any disagreement regarding eligibility was discussed with 2 additional authors (DW and HC) until consensus was reached. Among the included studies, antidepressant treatments were categorized by pharmacological class, and microbiota-related bacterial species or strains were standardized at the genus level. For multispecies probiotic formulations, individual species or strains were collapsed to their corresponding bacterial genera. Multiple strains belonging to the same genus within a single study were counted as 1 genus-level occurrence, and each independent study contributed a maximum of 1 count to each antidepressant class-genus pair. The frequency of each class-genus combination was then summarized and visualized using a bubble plot, with bubble size and color representing the number of unique studies examining each combination.

## 3. Results

### 3.1. Publication trends and global distribution

Between 2000 and 2026, a total of 1961 publications on antidepressant-gut microbiota research were included (Fig. 2A), of which 63.2% were articles and 36.8% were reviews. Annual publication output remained relatively low during the early years, increased gradually throughout the 2010s, and accelerated markedly after 2017. Accordingly, cumulative publication output also rose rapidly in recent years. Based on complete annual observations from 2000 to 2025, the third-order polynomial regression showed a strong fit to the publication trend (*R*^*2*^ = 0.9804), indicating sustained acceleration of research activity in this field (Fig. 2B). The 2026 publication count represents a partial-year observation through September 1, 2026 and was therefore excluded from the regression analysis.

**Figure 2.**
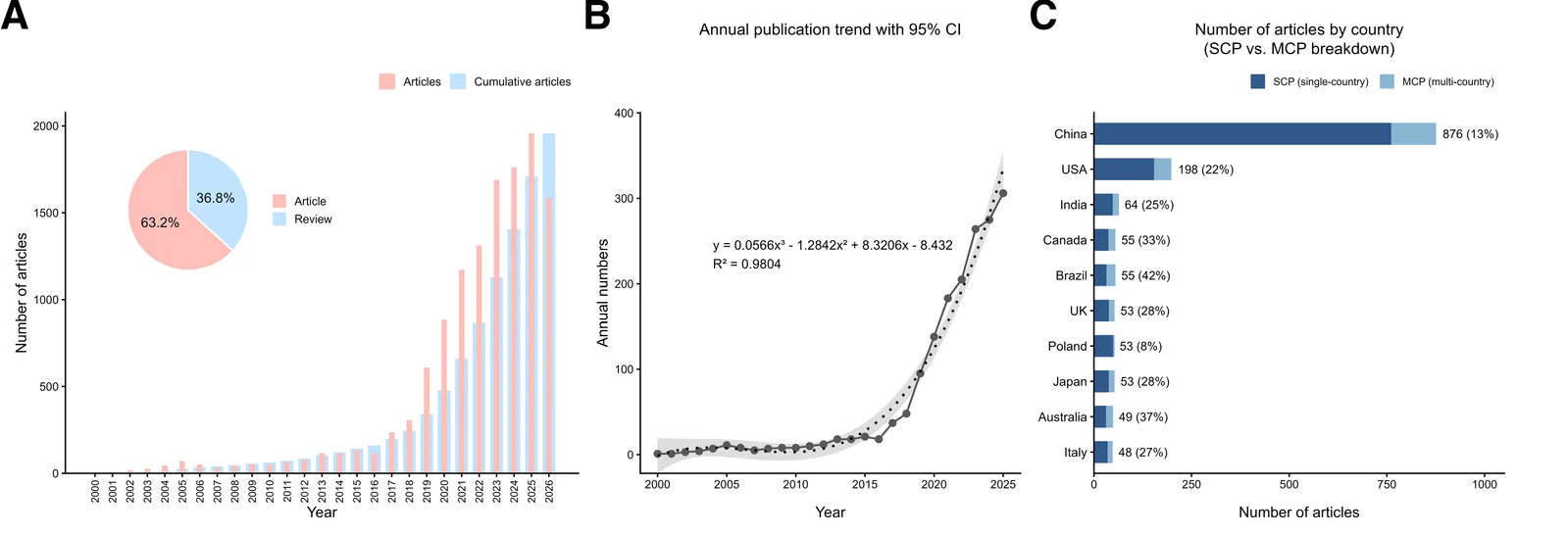
Publication trends and geographical distribution of antidepressant-gut microbiota research. (A) Annual and cumulative publication output and distribution of publication types from 2000 to 2026. (B) Annual publication trend with polynomial regression and 95% confidence interval. The 2026 publication count represents a partial-year observation through September 1, 2026 and was excluded from polynomial regression fitting. (C) Publication output of the leading countries, stratified by single-country publications (SCP) and multi-country publications (MCP). MCPs = multi-country publications, SCPs = single-country publications.

China contributed the largest number of publications (n = 876), followed by the United States (n = 198) and India (n = 64) (Fig. 2C). Despite its dominant publication output, China had a relatively modest proportion of MCPs (13%). In contrast, several countries with lower overall output exhibited higher proportions of international collaboration, particularly Brazil (42%), Australia (37%), and Canada (33%).

The international collaboration network showed extensive cross-country research connections, with China and the United States representing the most prominent nodes. The United States maintained broad collaborative links with China, the United Kingdom, Canada, Australia, Brazil, and several other countries, while relatively dense connections were also observed across Europe and Asia (Fig. 3). This pattern was broadly consistent with the MCP distribution in Figure 2C, as several countries with higher proportions of MCPs also showed more extensive international collaboration.

**Figure 3.**
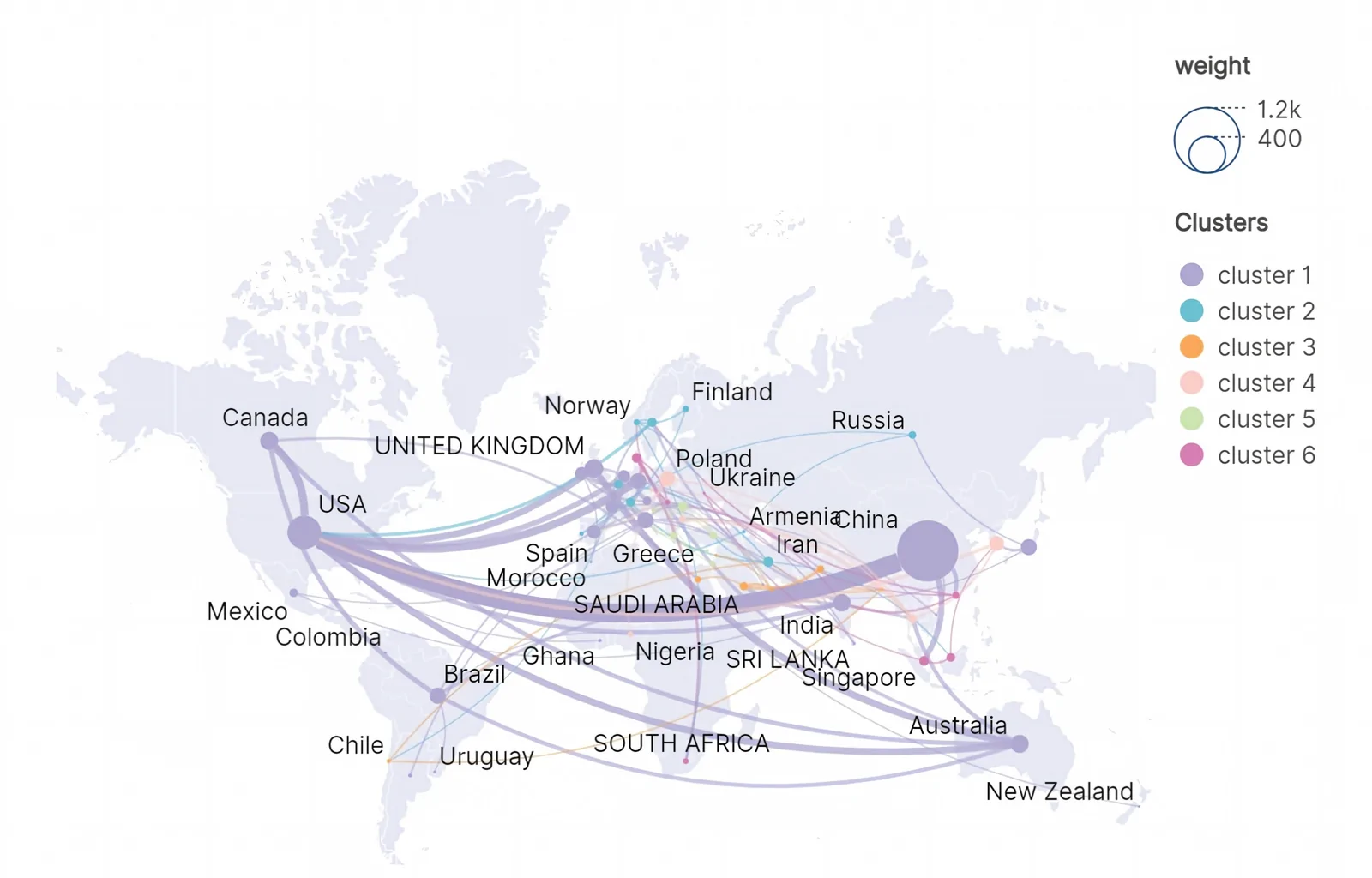
Cooperation among countries participating in antidepressant-gut microbiota research.

Table 4 presents the ten most productive authors in the antidepressant-gut microbiota field. Wang Y ranked first with 32 publications, followed by Hashimoto K (n = 29) and Qin XM (n = 26). Based on overall career-level H-index values retrieved from Web of Science on September 1, 2026, rather than H-indices calculated within the present bibliometric corpus, Cryan JF had the highest H-index (147), followed by Hashimoto K (101) and Chen W (84). The co-authorship network comprised 8 distinct collaborative clusters, and 9 of the ten most productive authors were represented among the visible network nodes. Prominent collaborative nodes included Wang Y, Hashimoto K, Qin XM, Wang G, Xie P, Chen W, and Cryan JF (Fig. 4).

**Table 4:** The top 10 authors in the field of antidepressant-gut microbiota.

Author	Country	Institution	H-index	Publications
Wang, Y	China	Shanghai University of Traditional Chinese Medicine	28	32
Hashimoto, K	Japan	Chiba University	101	29
Qin, XM	China	Shanxi University	41	26
Li, Y	China	Capital Medical University	50	22
Wang, G	China	Capital Medical University	59	20
Xie, P	China	Chongqing Medical University	64	19
Zhang, Y	China	Southwest University	12	18
Liu, Y	China	Chengdu University of Traditional Chinese Medicine	10	17
Chen, W	China	Jiangnan University	84	16
Cryan, JF	Ireland	University College Cork	147	16

**Figure 4.**
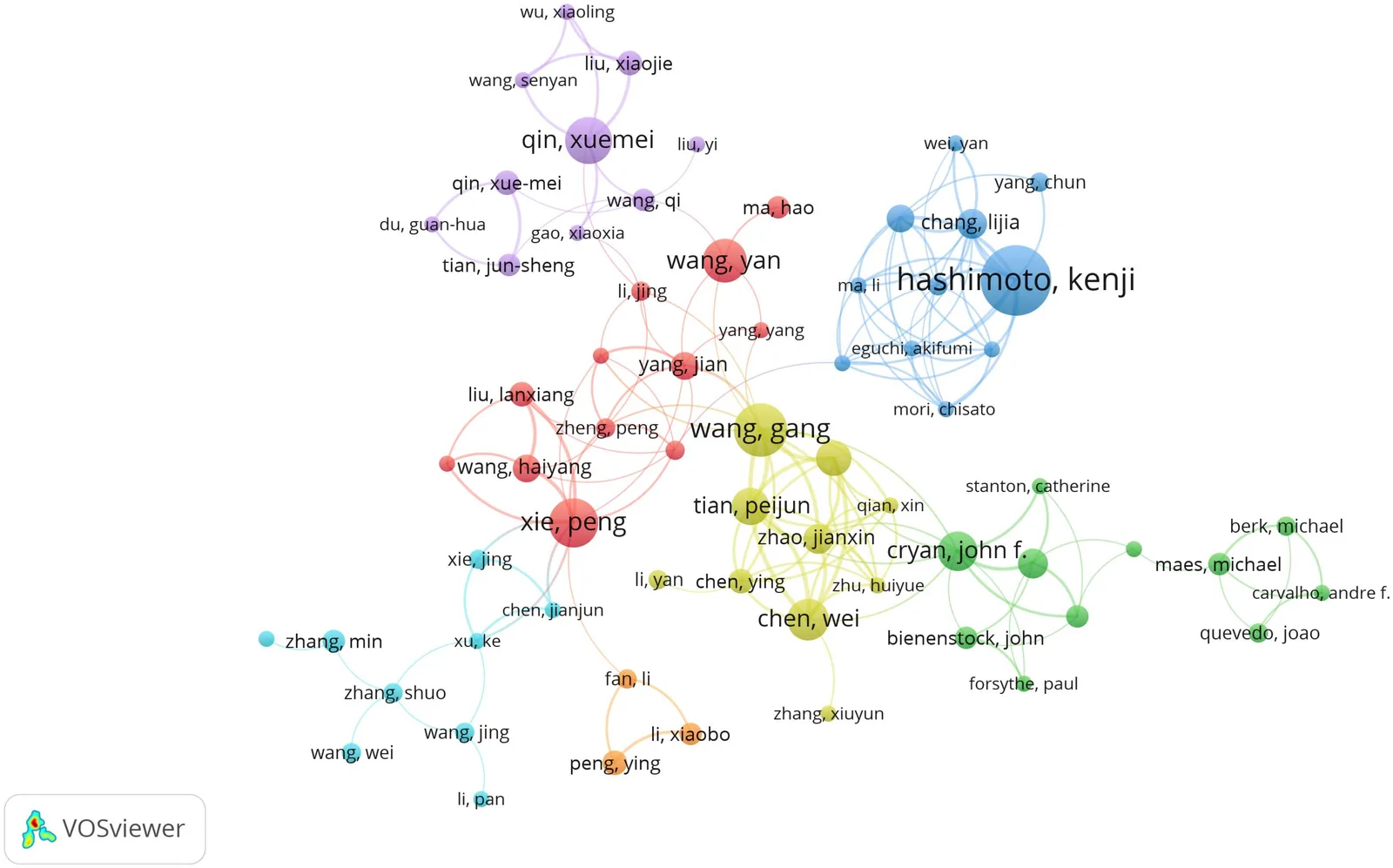
Author collaboration network in the antidepressant-gut microbiota field.

### 3.2. Research hotspots and thematic evolution

The cumulative keyword density map provides an overview of the major research topics in antidepressant-gut microbiota research (Fig. 5). “Depression” occupied the most prominent position and showed the highest concentration of surrounding keywords, accompanied by other frequently occurring terms such as “gut microbiota,” “intestinal flora,” “human,” “anxiety,” “brain,” and “antidepressant agent.” Mechanism and intervention-related terms, including “brain-gut axis,” “metabolites,” “psychobiotics,” “Lactobacillus,” and “stress,” were also widely represented.

**Figure 5.**
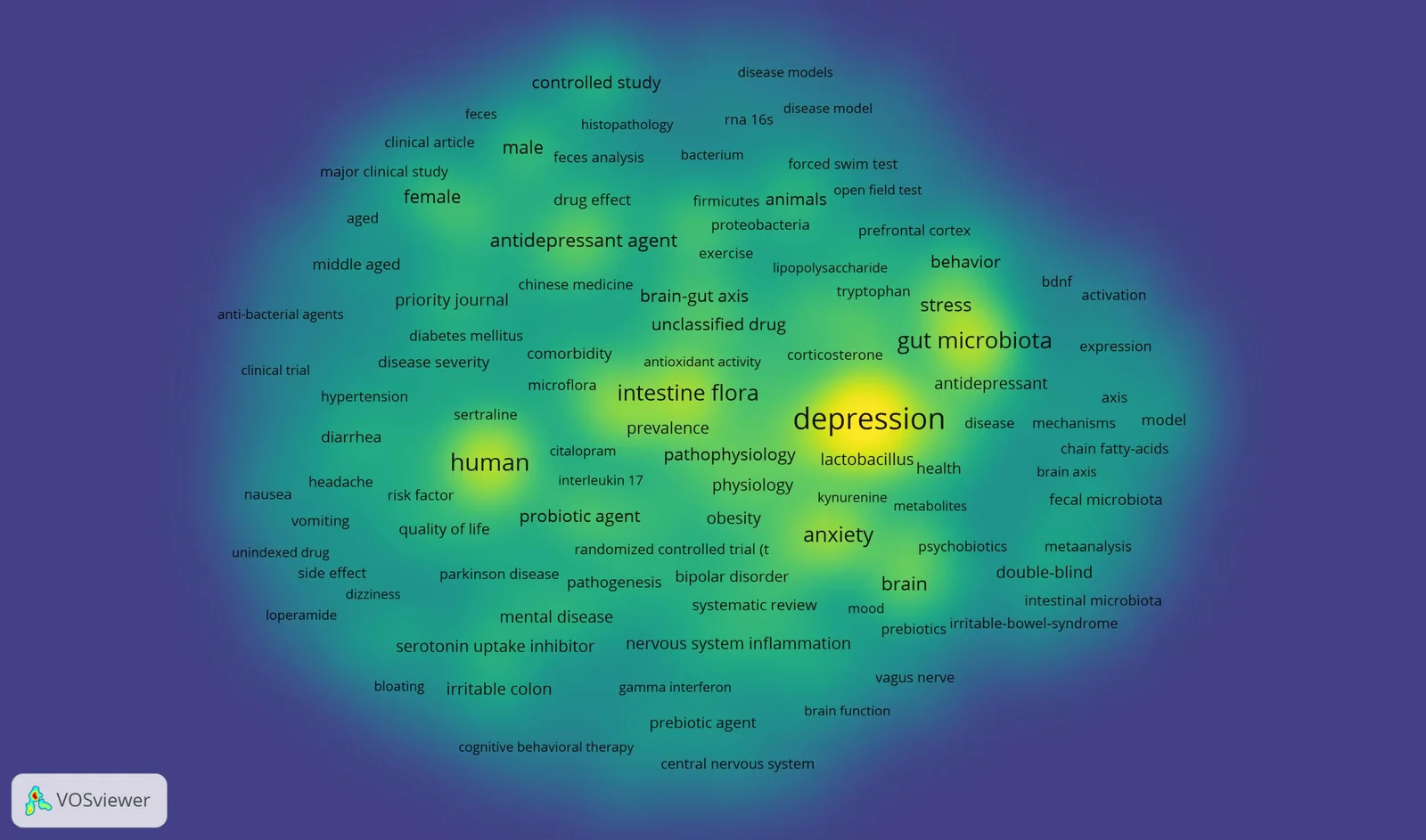
Cumulative keyword density map of antidepressant-gut microbiota research.

Keyword co-occurrence analysis further revealed the thematic structure of antidepressant-gut microbiota research (Fig. 6A). The VOSviewer network identified 4 interconnected clusters. The green cluster was centered on depression, gut microbiota, anxiety, stress, and brain, reflecting a major theme focused on depression-microbiota interactions and gut-brain communication. The yellow cluster was characterized by terms such as intestinal flora, brain-gut axis, tryptophan, lipopolysaccharide, and animal-related terms, indicating a strong emphasis on biological mechanisms and experimental research. The pink cluster mainly contained human and treatment-related terms, including anti-bacteria agents, side effects, clinical characteristics, and quality of life, whereas the blue cluster was more closely associated with nervous system inflammation, brain function, and related neurobiological processes. Within the network, “depression” occupied the most central position (occurrences = 695, total link strength [TLS] = 5904), followed by “gut microbiota” (TLS = 4420), “stress” (TLS = 2003), and “inflammation” (TLS = 1930). Other high-frequency keywords included microbiota (occurrences = 188, TLS = 1691), gut-brain axis (occurrences = 175, TLS = 1687), and brain (occurrences = 164, TLS = 1562). CiteSpace clustering provided a complementary view of the thematic structure and identified 7 major clusters: #0 neuroprotection, #1 probiotics, #2 gut microbiota, #3 drug industry, #4 irritable bowel syndrome, #5 female, and #6 gut-brain axis (Fig. 6B).

**Figure 6.**
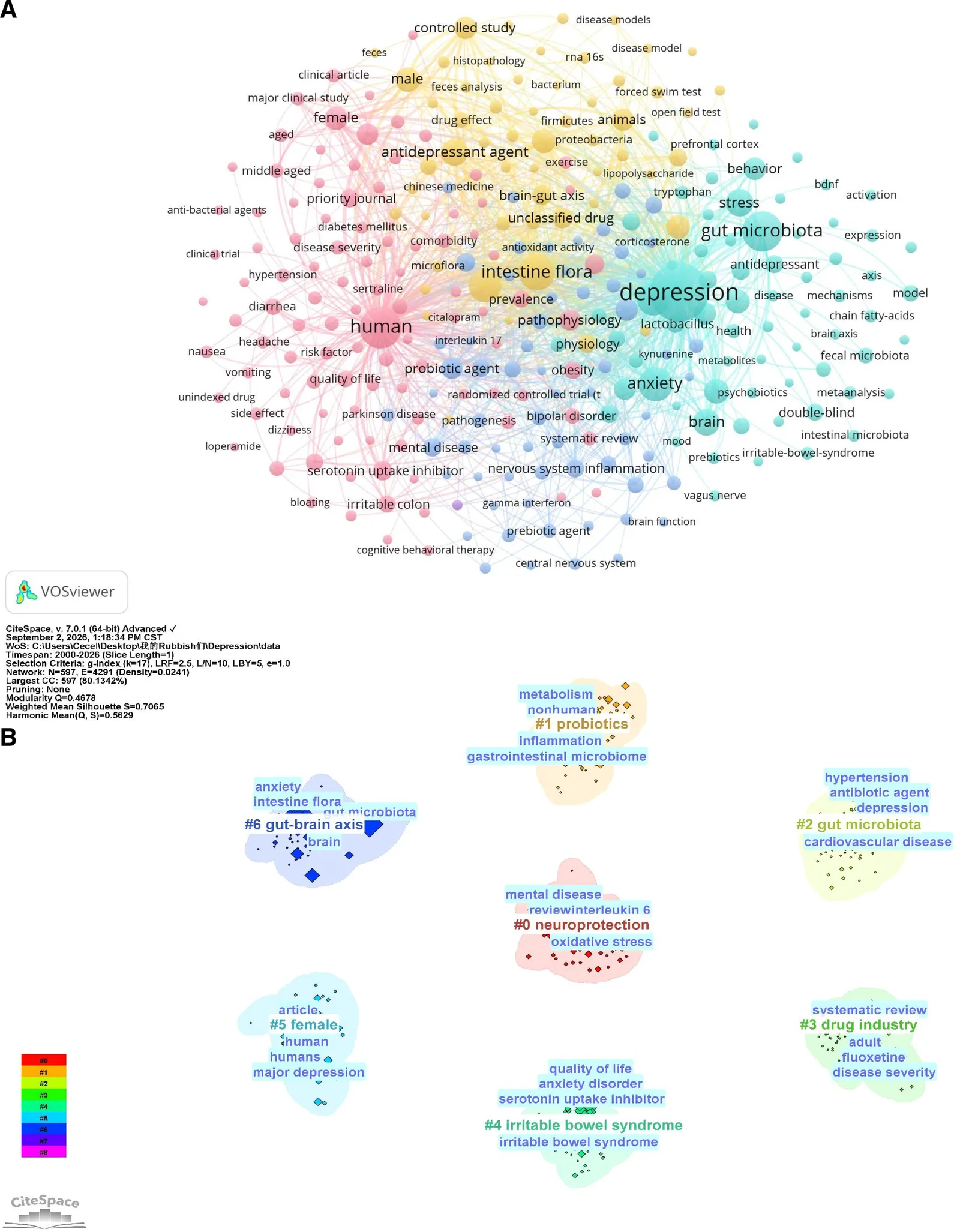
Visualization of keyword co-occurrence and thematic clustering in antidepressant-gut microbiota research. (A) Keyword co-occurrence network generated using VOSviewer, illustrating the relationships among major research topics. (B) Keyword clustering map generated using CiteSpace, showing the major thematic clusters within the field. The keyword co-occurrence network was constructed using author keywords and terms extracted from titles and abstracts. Node size represents occurrence frequency, and link thickness indicates co-occurrence strength.

The thematic evolution analysis illustrated how the research focus of this field has changed over time (Fig. 7A). During 2000 to 2007, research themes were relatively concentrated and were primarily centered on irritable bowel syndrome, suggesting that early studies may have mainly focused on gastrointestinal disorders and their links with psychological or emotional factors. From 2008 to 2014, the thematic scope expanded substantially, with the emergence of depression, stress, diabetes, constipation, treatment, and xiaoyaosan, indicating a gradual shift from a single gastrointestinal disorder toward a broader range of disease conditions and therapeutic approaches. Between 2015 and 2020, the field entered a period of rapid thematic diversification. On 1 hand, keywords such as probiotics, microbiota, and diet highlighted the growing importance of gut microbial composition and its modulators. On the other hand, the emergence of gut-brain axis, neurogenesis, and inflammation reflected increasing interest in the neural, immune, and biological mechanisms linking the gut microbiota to brain function. During 2021 to 2026, research themes became increasingly concentrated around gut-brain axis, depression, antidepressants, and neuroinflammation. Notably, the themes of gut-brain axis, probiotics, and microbiota, which had emerged prominently in the previous period, continued to show strong connections and increasingly converged with research on depression, antidepressant treatment, and neuroinflammatory mechanisms.

**Figure 7.**
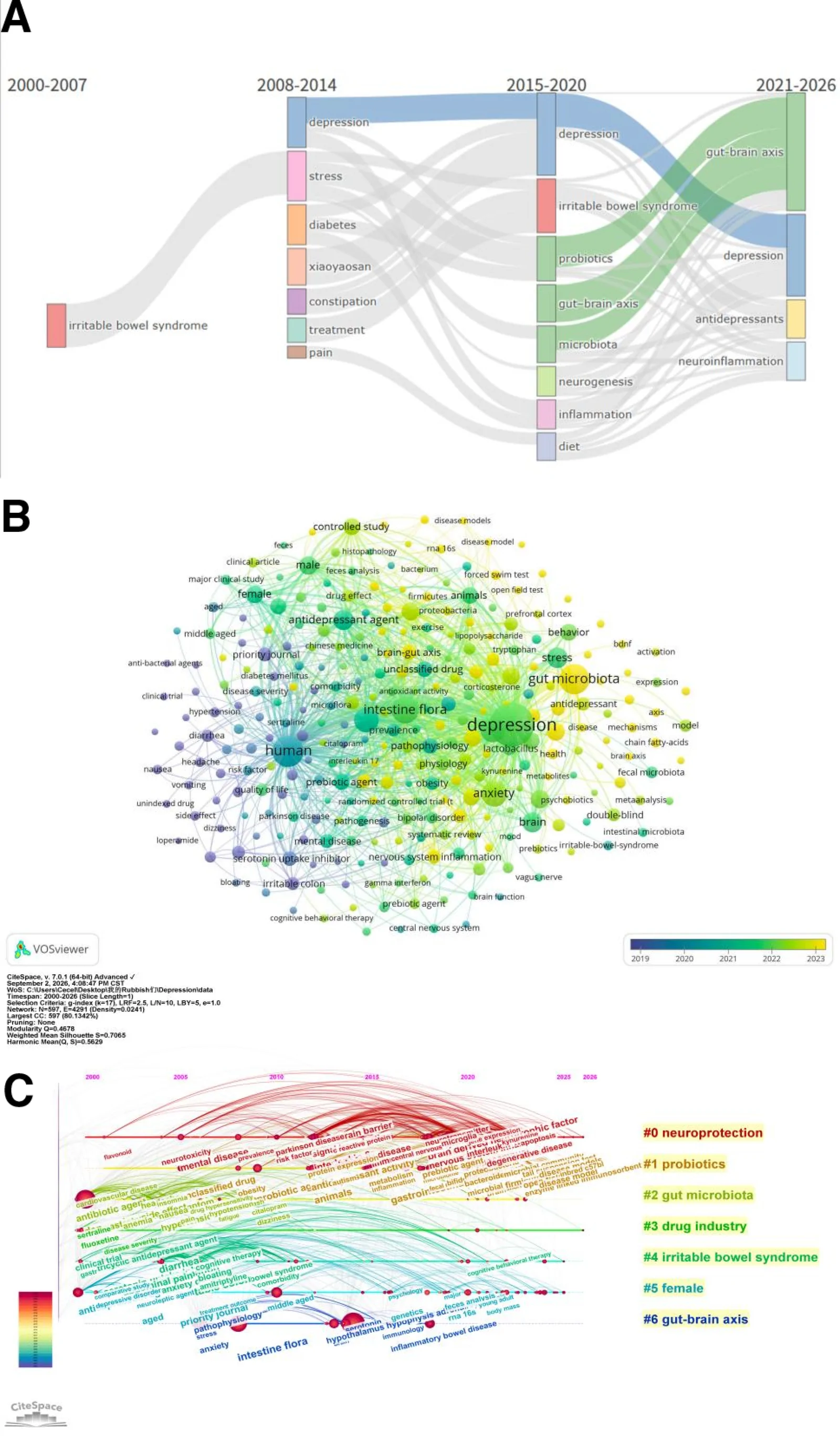
Temporal evolution of research themes and keywords in antidepressant-gut microbiota research. (A) Thematic evolution across 4 time periods (2000-2007, 2008-2014, 2015-2020, and 2021-2026). (B) Keyword overlay visualization generated using VOSviewer, with colors representing the average publication year of keywords. (C) Timeline visualization of major keyword clusters generated using CiteSpace, illustrating their temporal distribution and evolution.

The temporal distribution of keywords further revealed the dynamic evolution of research hotspots in this field. The VOSviewer overlay visualization showed that earlier studies were mainly associated with human, female, intestinal flora, and clinical or disease-related themes, whereas more recent research increasingly focused on depression, gut microbiota, gut-brain axis, and probiotics (Fig. 7B). The CiteSpace timeline analysis identified 7 thematic clusters. Among them, #0 neuroprotection, #1 probiotics, #5 female, and #6 gut-brain axis exhibited relatively long temporal spans, whereas #2 gut microbiota, #3 drug industry, and #4 irritable bowel syndrome were predominantly active during earlier periods (Fig. 7C).

### 3.3. Citation impact and research influence

The dual-map overlay analysis illustrated the disciplinary distribution of citing journals (left) and cited journals (right) (Fig. 8). The major citation trajectories showed that research published in Molecular, Biology, and Genetics journals primarily drew upon knowledge from Molecular, Biology and Genetics, Health, Nursing, and Medicine, Psychology, Education, and Social. These citation pathways reflect the multidisciplinary knowledge base underlying antidepressant-gut microbiota research.

**Figure 8.**
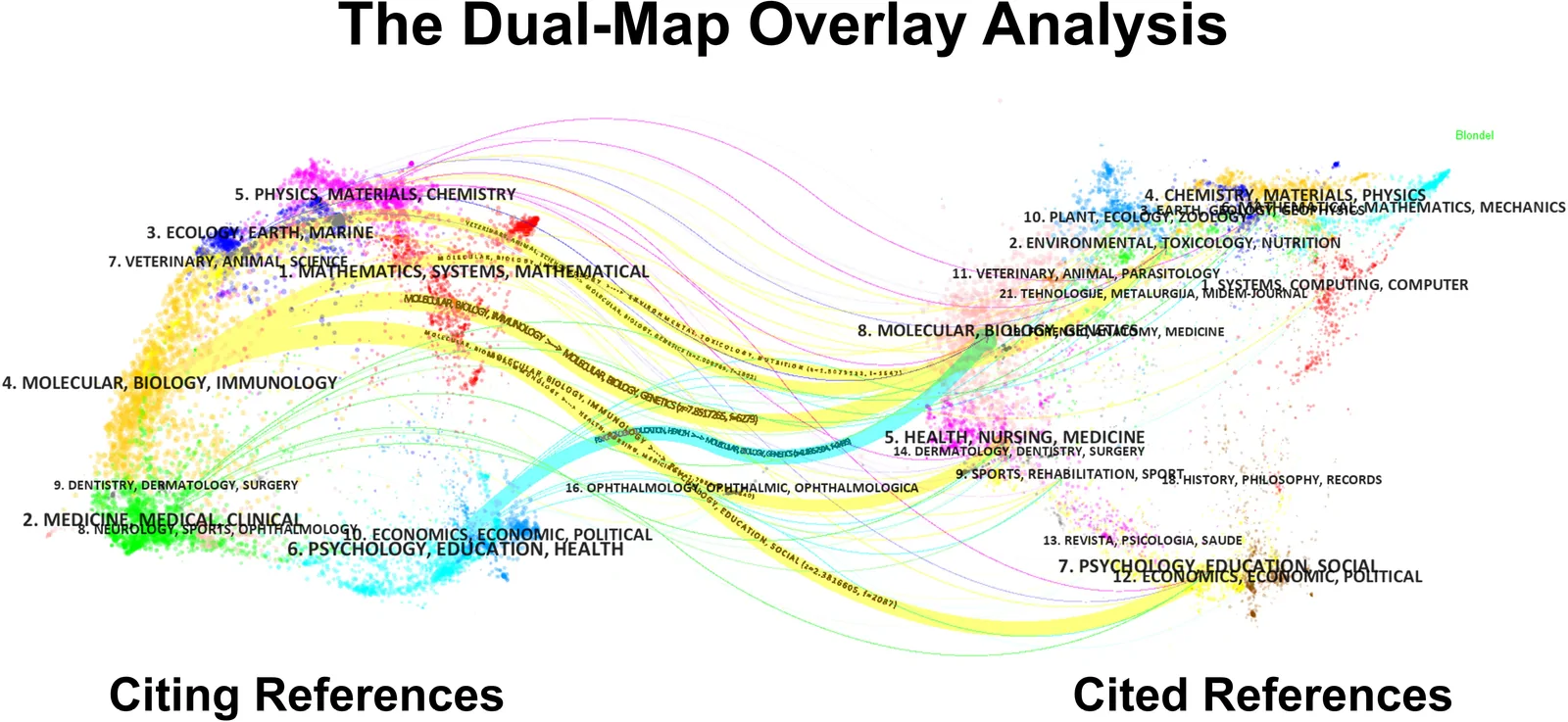
Dual-overlay map in the field of antidepressant-gut microbiota research.

The ten most-cited publications could be broadly categorized into 4 major themes ^[16–25]^ (Table 5). Four publications focused on probiotics or psychobiotics and their potential antidepressant effects,^[19,20,22,24]^ representing the most prominent theme among the highly cited literature. Three publications addressed the relationship between gut microbiota and depression, including alterations in microbial composition, neuroactive potential, and microbiota-derived metabolites.^[16,17,23]^ Two publications focused on the interplay between inflammation and depression, including inflammasome-related mechanisms.^[17,25]^ In addition, one highly cited study specifically investigated the microbiota-gut-brain axis by examining the anxiolytic and antidepressant-like effects of prebiotic intervention.^[21]^

**Table 5:** The top 10 globally cited publications in the field of antidepressant-gut microbiota research.

Rank	Title	Citation	First author	Journal	Yr
1	Altered fecal microbiota composition in patients with major depressive disorder	1834	Jiang, HY	Brain Behavior and Immunity	2015
2	The Bidirectional Relationship of Depression and Inflammation: Double Trouble	1812	Beurel, E	*Neuron*	2020
3	The neuroactive potential of the human gut microbiota in quality of life and depression	1517	Valles-Colomer, M	Nature Microbiology	2019
4	Psychobiotics: A Novel Class of Psychotropic	971	Dinan, TG	Biological Psychiatry	2013
5	The probiotic Bifidobacteria infantis: An assessment of potential antidepressant properties in the rat	769	Desbonnet, L	Journal of Psychiatric Research	2008
6	Targeting the Microbiota-Gut-Brain Axis: Prebiotics Have Anxiolytic and Antidepressant-like Effects and Reverse the Impact of Chronic Stress in Mice	751	Burokas, A	Biological Psychiatry	2017
7	Effects of the Probiotic Bifidobacterium Infantis in the Maternal Separation Model of Depression	750	Desbonnet, L	Neuroscience	2010
8	Gut microbiota and its metabolites in depression: from pathogenesis to treatment	567	Liu, LX	Ebiomedicine	2023
9	Administration of Lactobacillus Helveticus NS8 Improves Behavioral, Cognitive, and Biochemical Aberrations Caused by Chronic Restraint Stress	521	Liang, S	Neuroscience	2015
10	Inflammasome signaling affects anxiety- and depressive-like behavior and gut microbiome composition	462	Wong, ML	Molecular Psychiatry	2016

Citation burst analysis revealed a concentrated pattern of citation attention over time (Fig. 9). The strongest bursts emerged mainly after 2017, with Zheng *P* showing the highest burst strength (42.95), followed by Jiang HY (strength = 37.67) and Kelly JR (strength = 36.41). Notably, several burst references also appeared among the most frequently cited publications in the present dataset. Studies by Jiang HY, Wong ML, Burokas A, Liang S, and Liu LX were also ranked among the ten most-cited articles in Table 5, while Cryan JF, one of the most productive authors, also appeared among the burst references. More recently, the citation bursts of Liu LX and Radjabzadeh D persisted through 2026, indicating sustained citation attention to these studies.

**Figure 9.**
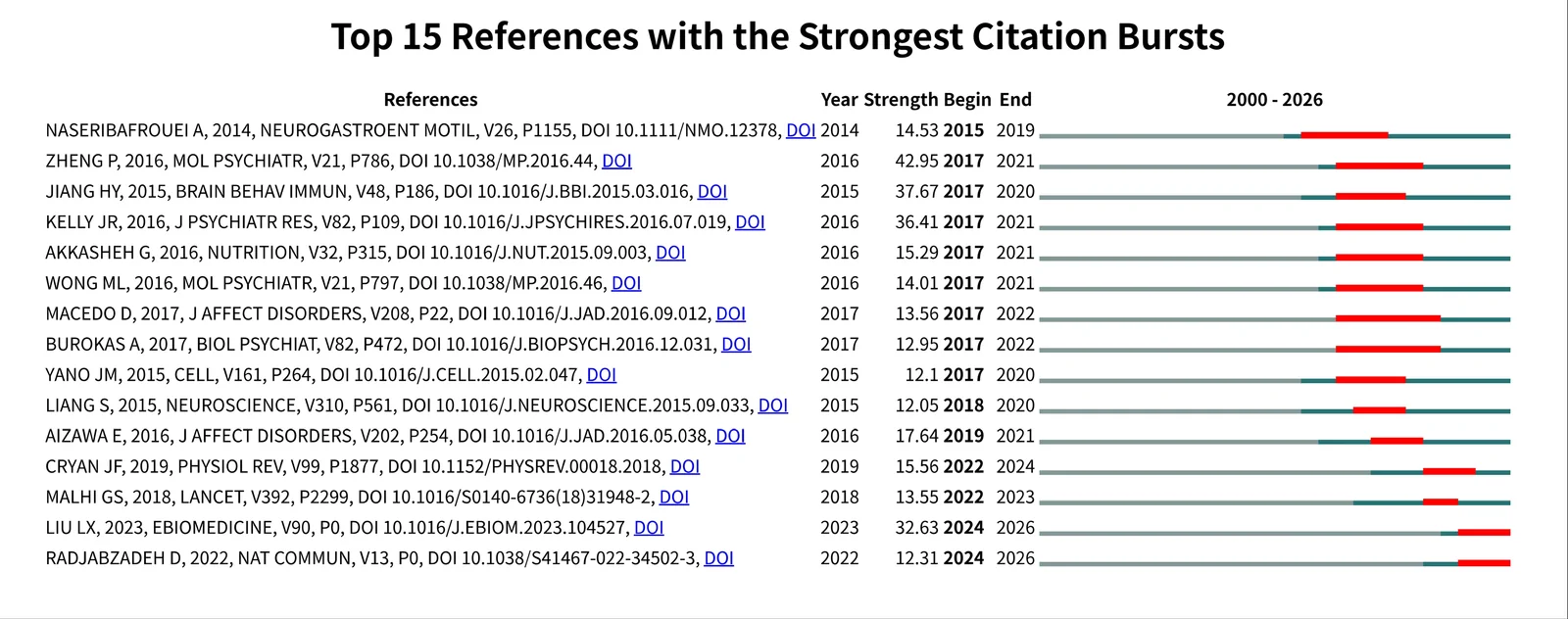
Citation analysis. Timeline chart showing top 15 references with the strongest citation bursts as horizontal bars across years.

### 3.4. Clinical evidence and translational landscape

To assess the clinical translation of antidepressant-gut microbiota research, we systematically evaluated 13 published clinical studies identified through PubMed. Detailed information on publication title, first author, journal, trial registration, study design, study population, antidepressant treatment, microbiota-related intervention, follow-up duration, and key findings is summarized in Table 6.^[26–38]^ Among the 13 included clinical studies, 11 were randomized controlled trials, most of which evaluated microbiota-related approaches as adjuncts to ongoing antidepressant treatment. Intervention periods ranged from 4 to 12 weeks, with approximately 8 weeks being the most common duration. Probiotic-based interventions were examined in 10 studies and represented the predominant microbiota-targeted strategy, particularly formulations containing Lactobacillus and Bifidobacterium species. Other approaches included fecal microbiota transplantation, Danzhi-xiaoyao-San as an adjunctive intervention, and assessment of the intestinal microbiota as a potential predictor of antidepressant treatment response.

**Table 6:** Clinical translational evidence for antidepressant-gut microbiota interactions.

Study	Registration/publication	Design	Population	Antidepressant treatment	Microbiota-related intervention	Follow-up	Key findings
Adjunctive fecal microbiota transplantation for major depressive disorder: A randomized, double-blind, placebo-controlled trial^[26]^ Jing Li, et al *Cell Host Microbe*	PMID: 42309058 DOI: 10.1016/j.chom.2026.05.017	Randomized, double-blind, placebo-controlled trial	60 patients with major depressive disorder (MDD)	escitalopram	Fecal microbiota transplantation (FMT) capsules	8 wk	FMT produced greater reductions in Hamilton Depression Rating Scale, 17-item version (HAMD-17) scores at wk 2 and 8. Multi-omics analyses show durable donor microbial engraftment and enrichment of beneficial Lachnospiraceae and Oscillospiraceae taxa. Microbial remodeling is accompanied by an increase in serum bile acids that correlate with the alleviation of depressive symptoms.
Probiotic Lactobacillus plantarum 299v supplementation in patients with major depression in a double-blind, randomized, placebo-controlled trial: A metabolomics study^[27]^ Joanna Godzien, et al *J Affect Disord*	NCT02469545 PMID: 39271063 DOI: 10.1016/j.jad.2024.09.058	Randomized, double-blind, placebo-controlled trial	60 patients with major depressive disorder (MDD)	Selective serotonin reuptake inhibitors (SSRIs)	Lactobacillus plantarum 299v (LP299v)	8 wk	Supplementation of SSRI treatment with LP299v intensified the reduction of long-chain acylcarnitines, potentially indicating improved mitochondrial function. LP299v supplementation reduced N-acyl taurines more than 4 times compared to the placebo, suggesting a substantial impact on restoring biochemical balance. The LP299v-supplemented group showed increased levels of oxidized glycerophosphocholine (oxPC). Additionally, LP299v supplementation led to higher levels of sphingomyelins, L-histidine, D-valine, and p-cresol.
The intestinal microbiota as a predictor for antidepressant treatment outcome in geriatric depression: a prospective pilot study^[28]^ S Melanie Lee, et al *Int Psychogeriatr*	NCT02466958 PMID: 33757609 DOI: 10.1017/S1041610221000120	randomized placebo-controlled trial	17 patients aged > 60 years with major depressive disorder (MDD)	Levomilacipran (LVM) or placebo	None (intestinal microbiota was assessed as a predictor)	12 wk	Baseline enrichment of Faecalibacterium, Agathobacter and Roseburia relative to a reference frame was associated with treatment outcome of remission. Differential abundance analysis revealed significant genus level changes from baseline to post-treatment in remitters, but not in non-remitters.
Efficacy of Adjunct PRObiotics as Compared to the Standard Care in Moderate Unipolar Depression Among Geriatric Patients: A Randomized Double-Blind Placebo-Controlled Pilot Multi-Center Trial (PRODG)^[29]^ Preeti Sinha, et al *J Am Geriatr Soc*	PMID: 42304935 DOI: 10.1111/jgs.70530	randomized double-blind placebo-controlled pilot multi-center trial	58 participants (≥ 60 yr) with moderate depression	standard antidepressant care (low-dose benzodiazepines for concomitant anxiolytics patients)	Lactobacillus helveticus and Bifidobacterium longum	12 wk	Overall scores were lower in the probiotic group compared with the placebo group for both MADRS (F = 12.7, *P* = .001) and GAD-7 (F = 10.7, *P* = .002). Quality-of-life domains improved markedly (all F > 100, *P* < .001) without additional benefit from probiotics. The probiotic group also had a significantly higher serum BDNF level and increased fecal abundance of supplemented strains vs the placebo group.
The pharmacological actions of Danzhi-xiaoyao-San on depression involve lysophosphatidic acid and microbiota-gut-brain axis: novel insights from a systems pharmacology analysis of a double-blind, randomized, placebo-controlled clinical trial^[30]^ Xiuqing Zhu, et al *J Biomol Struct Dyn*	PMID: 37632305 DOI: 10.1080/07391102.2023.2251067	randomized, double-blind, placebo-controlled trial	32 depressed patients receiving selective serotonin reuptake inhibitors (SSRIs)	selective serotonin reuptake inhibitors (SSRIs)	Danzhi-xiaoyao-San (DZXYS)	4 wk	DZXYS combined with SSRIs improved depressive symptoms more than SSRIs alone. 16S rRNA sequencing identified 32 differential microbial taxa associated with DZXYS treatment. Several taxa were correlated with lysophosphatidic acid-related metabolites, supporting modulation of the microbiota–gut–brain axis as a potential mechanism of adjunctive antidepressant efficacy.
Clostridium butyricum MIYAIRI 588 as Adjunctive Therapy for Treatment-Resistant Major Depressive Disorder: A Prospective Open-Label Trial^[31]^ Tsuyoshi Miyaoka, et al *Clin Neuropharmacol*	PMID: 30234616 DOI: 10.1097/WNF.0000000000000299	Prospective open-label trial	40 hospitalized adults with treatment-resistant major depressive disorder (TRD)	Antidepressants (flvoxamine, paroxetine, escitalopram, duroxetine, and sertraline)	Clostridium butyricum MIYAIRI 588 (CBM588)	8 wk	Adjunctive CBM588 significantly improved depressive symptoms when combined with ongoing antidepressant therapy. Approximately 70% of patients showed a treatment response and 35% achieved remission, with no serious adverse events reported.
A combination of probiotics and magnesium orotate attenuate depression in a small SSRI resistant cohort: an intestinal anti-inflammatory response is suggested^[32]^ Matthew Bambling, et al *Inflammopharmacology*	PMID: 28155119 DOI: 10.1007/s10787-017-0311-x	Prospective pilot study	12 patients with treatment-resistant depression receiving SSRIs	selective serotonin reuptake inhibitors (SSRIs)	probiotics/magnesium orotate formulation adjuvant	8 wk (16 wk follow-up)	At the end of an 8-week intervention mean changes for depression scores and quality of life in the group was clinically significantly improved (*P* < .001) with all but 4 participants experiencing a benefit. An intestinal anti-inflammatory response was suggested. At 16-weeks follow-up while still on SSRI medications, the group had relapsed after cessation of the test intervention.
Interleukin-6 Gene Expression Changes after a 4-Week Intake of a Multispecies Probiotic in Major Depressive Disorder-Preliminary Results of the PROVIT Study^[33]^ Alexandra Reiter, et al *Nutrients*	NCT03300440 PMID: 32858844 DOI: 10.3390/nu12092575	monocentric, randomized, placebo-controlled trial (preliminary PROVIT analysis)	61 hospitalized patients with major depressive disorder (MDD)	Antidepressant treatment within the PROVIT clinical setting (Glutamatergic antidepressants, Melatonin-like antidepressants, Noradrenergic and specific serotonergic antidepressants, Norepinephrine-dopamine reuptake inhibitor (NDRI), Selective serotonin reuptake inhibitors (SSRIs), Serotonin-norepinephrine reuptake inhibitors (SNRIs), Tri- and tetracyclic antidepressants (TZA))	Multispecies-probiotic “OMNi-BiOTiC® STRESS Repair” (Lactobacillus casei W56, Lactobacillus acidophilus W22, Lactobacillus paracasei W20, Bifidobacterium lactis W51, Lactobacillus salivarius W24, Lactococcus lactis W19, Bifidobacterium lactis W52, Lactobacillus plantarum W62, Bifidobacterium bifidum W23)	4 wk	In IL-6 we found no significant main effects for group (F (1,44) = 1.33, p = ns) nor time (F (1,44) = 0.00, *p *= ns), but interaction was significant (F(1,44) = 5.67, *P* < .05). The intervention group showed decreasing IL-6 gene expression levels while the placebo group showed increasing gene expression levels of IL-6.
Acceptability, Tolerability, and Estimates of Putative Treatment Effects of Probiotics as Adjunctive Treatment in Patients With Depression: A Randomized Clinical Trial^[34]^ Viktoriya L Nikolova, et al *JAMA Psychiatry*	NCT03893162 PMID: 37314797 DOI: 10.1001/jamapsychiatry.2023.1817	Single-center, double-blind, placebo-controlled pilot randomized clinical trial	49 outpatients with a primary diagnosis of MDD	Ongoing antidepressant medication	Probiotic (Bacillus subtilis, Bifidobacterium bifidum, Bifidobacterium breve, Bifidobacterium infantis, Bifidobacterium longum, Lactobacillus acidophilus, Lactobacillus delbrueckii subsp bulgaricus, Lactobacillus casei, Lactobacillus plantarum, Lactobacillus rhamnosus, Lactobacillus helveticus, Lactobacillus salivarius, Lactococcus lactis, and Streptococcus thermophilus)	8 wk	Standardized effect sizes (SES) from linear mixed models demonstrated that the probiotic group attained greater improvements in depressive symptoms according to HAMD-17 scores (wk 4: SES, 0.70; 95% CI, 0.01-0.98) and IDS Self Report scores (week 8: SES, 0.64; 95% CI, 0.03–0.87) as well as greater improvements in anxiety symptoms according to HAMA scores (wk 4: SES, 0.67; 95% CI, 0–0.95; wk 8: SES, 0.79; 95% CI, 0.06–1.05), but not GAD-7 scores (wk 4: SES, 0.57; 95% CI, −0.01 to 0.82; week 8: SES, 0.32; 95% CI, −0.19 to 0.65), compared with the placebo group.
Effect of probiotic and prebiotic vs placebo on psychological outcomes in patients with major depressive disorder: A randomized clinical trial^[35]^ Asma Kazemi, et al *Clin Nutr*	PMID: 29731182 DOI: 10.1016/j.clnu.2018.04.010	Randomized, double-blind, 3-arm parallel design, placebo-controlled trial	81 patients with major depressive disorder (MDD)	Sertraline, fluoxetine, citalopram or amitriptyline	Probiotic (L. helveticus R0052 and B. longum R0175 (CNCM strain I-3470) bacteria)	8 wk	From baseline to 8 wk, probiotic supplementation resulted in a significant decrease in BDI score (17.39-9.1) compared to the placebo (18.18-15.55) and prebiotic (19.72–14.14) supplementation (*P* = .042). Inter-group comparison indicated no significant differences among the groups in terms of serum kynurenine/tryptophan ratio and tryptophan/BCAAs ratio. However, the kynurenine/tryptophan ratio decreased significantly in the probiotic group compared to the placebo group after adjusting for serum isoleucine (*P* = .048). In addition, the tryptophan/isoleucine ratio increased significantly in the probiotic group when compared to the placebo group (*P* = .023).
Bifidobacterium breve CCFM1025 attenuates major depression disorder via regulating gut microbiome and tryptophan metabolism: A randomized clinical trial^[36]^ Peijun Tian, et al *Brain Behav Immun*	PMID: 34875345 DOI: 10.1016/j.bbi.2021.11.023	Randomized, double-blind, placebo-controlled trial	45 patients with MDD	Ongoing antidepressant medication (SSRIs)	Freeze-dried Bifidobacterium breve CCFM1025 powder	8 wk	CCFM1025 showed a better antidepressant-like effect than placebo, based on the HDRS-24 (placebo: M = 6.44, SD = 5.44; CCFM1025: M = 10.40, SD = 6.85; t(43) = 2.163, *P* = .036, d = 0.640) and MADRS (placebo: M = 4.92, SD = 7.15; CCFM1025: M = 9.60, SD = 7.37; t(43) = 2.152, *P* = .037, d = 0.645) evaluation.
The effects of Lactobacillus plantarum PS128 in patients with major depressive disorder: an eight-week double-blind, placebo-controlled study^[37]^ Shih-Kai Kevin Lin, et al *Asian J Psychiatr*	PMID: 39232392 DOI: 10.1016/j.ajp.2024.104210	randomized, double-blind, placebo-controlled trial	32 patients with MDD	ongoing antidepressant medication	Lactobacillus plantarum PS128 (PS128)	8 wk	After 8 wk, depressive symptoms improved significantly in both groups, but no significant between-group difference was observed. PS128 supplementation did not significantly affect inflammatory or gut permeability biomarkers, nor did it produce significant changes in overall gut microbiota composition compared with placebo.
Metabolic Status Influences Probiotic Efficacy for Depression-PRO-DEMET Randomized Clinical Trial Results^[38]^ Oliwia Gawlik-Kotelnicka, et al *Nutrients*	NCT04756544 PMID: 38732635 DOI: 10.3390/nu16091389	parallel-group, prospective, randomized, double-blind, controlled design	116 participants with depression	ongoing antidepressant medication	Lactobacillus helveticus Rosell-52 and Bifidobacterium longum Rosell-175	8 wk	There was no advantage of probiotics usage over placebo in the depression score overall (PRO vs PLC: F(1.92) = 0.58; *P* = .45). However, we found a higher rate of minimum clinically important differences in patients supplemented with probiotics than those allocated to placebo generally (74.5 vs. 53.5%; X2(1,n = 94) = 4.53; *P* = .03; NNT = 4.03), as well as in the antidepressant-treated subgroup.

Clinical findings were heterogeneous. Several studies reported greater improvements in depressive symptoms following adjunctive probiotic or microbiota-targeted interventions, whereas others found no significant overall advantage over placebo.^[37,38]^ Beyond symptomatic outcomes, several trials incorporated microbiome, metabolomic, inflammatory, or neurobiological measures, including microbial composition, tryptophan metabolism, interleukin-6expression, BDNF, and circulating metabolites. One study further examined baseline intestinal microbiota as a potential predictor of antidepressant treatment outcome.^[28]^

The distribution of bacterial genera across antidepressant treatment categories was visualized in Figure 10. The most frequently represented combinations involved unspecified or mixed antidepressant treatment with Lactobacillus, Bifidobacterium, and Bacillus. These genera were also frequently represented in studies involving selective serotonin reuptake inhibitors, particularly Lactobacillus and Bifidobacterium. TCA-based treatment was mainly associated with Lactobacillus and Bifidobacterium, whereas comparatively few studies involved serotonin-norepinephrine reuptake inhibitors or other antidepressant classes. Overall, Lactobacillus and Bifidobacterium were the most consistently represented bacterial genera across different antidepressant treatment categories.

**Figure 10.**
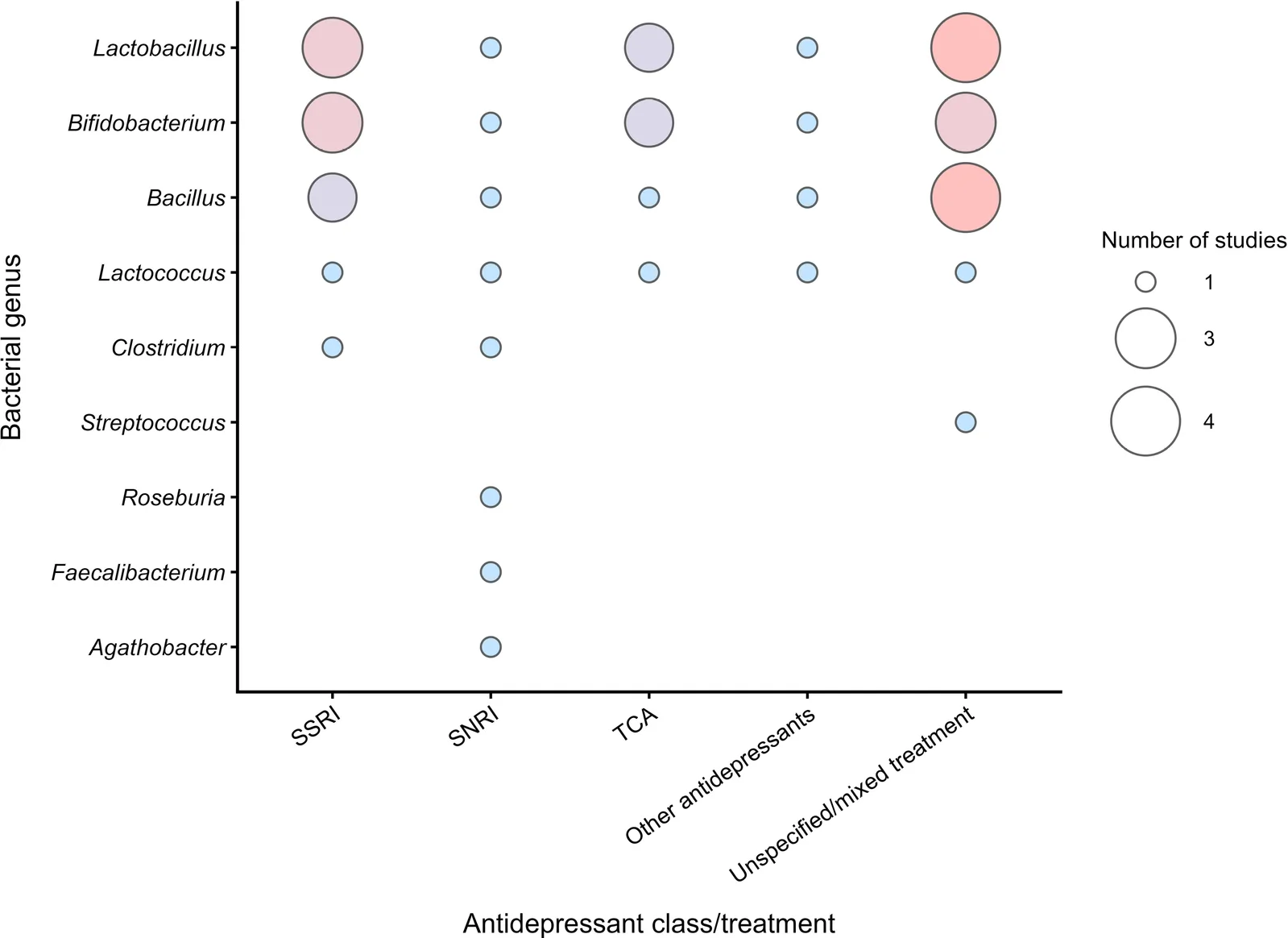
Distribution of bacterial genera across antidepressant treatment categories in the included clinical studies. Bubble size and color indicate the number of unique studies examining each antidepressant class-genus combination, with larger and pinker bubbles representing more studies and smaller and bluer bubbles representing fewer studies. SNRIs = serotonin-norepinephrine reuptake inhibitors, SSRIs = selective serotonin reuptake inhibitors, TCAs = tricyclic antidepressants.

## 4. Discussion

This study provides a comprehensive overview of the evolving landscape of antidepressant-gut microbiota research. The bibliometric analyses characterized the growth, collaboration patterns, and thematic evolution of the field, whereas the separate clinical translational assessment summarized the current human intervention evidence. The field expanded rapidly after 2017, with China contributing the largest publication output and the United States showing relatively broad international collaboration. Research themes progressively shifted toward the gut-brain axis, probiotics, inflammation, and antidepressant-related mechanisms. In contrast, the clinical translational assessment showed that human intervention evidence has begun to emerge but remains limited and heterogeneous.

The rapid increase in publications after 2017 may partly reflect advances in microbiome sequencing and multi-omics technologies, which enabled increasingly detailed characterization of microbial composition and host-microbiome interactions. In 2014, Naseribafrouei et al ^[39]^ used 16S rRNA sequencing to identify distinct fecal microbial signatures associated with depression, including alterations in Oscillibacter and Alistipes, providing early evidence linking specific gut microbial taxa to depressive disorders. Subsequently, Zheng et al ^[40]^ provided the first experimental evidence that alterations in the gut microbiota could causally contribute to depression-like behaviors. Fecal microbiota transplantation from patients with major depressive disorder induced depression-like behaviors in recipient mice, accompanied by disturbances in carbohydrate and amino acid metabolism. Importantly, consistent metabolic alterations were observed across fecal and serum samples and in the hippocampus, where disrupted carbohydrate metabolism involving glucose, lactose, and malate and amino acid metabolism involving phenylalanine, N-acetyl-L-aspartate, glycine, and leucine were detected. These findings provided functional and metabolic evidence linking gut microbial alterations to changes in the brain, thereby strengthening the biological basis of the microbiota-gut-brain axis in depression. Notably, this study also showed the strongest citation burst in our analysis (strength =42.95). Together, technological advances and these influential studies may have facilitated the shift from descriptive microbial associations toward mechanistic investigation, coinciding with the marked acceleration of research output after 2017.

Our thematic analyses revealed a parallel shift in the scientific focus of the field. Early research was predominantly centered on gastrointestinal disorders, particularly irritable bowel syndrome, whereas subsequent studies increasingly incorporated depression, stress, and therapeutic interventions. From 2015 onward, the emergence of microbiota, probiotics, the gut-brain axis, inflammation, and neurogenesis marked a transition from largely descriptive and associative research toward mechanistic investigation. More recently, these themes have increasingly converged around depression, antidepressants, and neuroinflammation, suggesting that elucidating the biological pathways linking the gut microbiota to brain function and antidepressant response has become a central research focus.

The microbiota-gut-brain axis provides the principal biological framework for understanding these interactions. It comprises bidirectional communication between the gastrointestinal tract and the central nervous system through immune-inflammatory, metabolic, neuroendocrine, and neural signaling. Among these pathways, immune-inflammatory signaling has emerged as one of the most extensively investigated mechanisms linking gut microbial dysbiosis with depression.Stress-related disruption of the gut microbiota may alter the production of microbial bioactive products and microbial-associated molecular patterns,^[41]^ which can be recognized by host pattern-recognition receptors, including Toll-like receptors and Nucleotide-binding Oligomerization Domain -like receptors such as the Nucleotide-binding Oligomerization Domain -like receptor family pyrin domain-containing 3 inflammasome^[42,43]^ Activation of these innate immune pathways promotes the release of pro-inflammatory cytokines, which may further increase intestinal permeability and impair blood-brain barrier integrity.^[44]^ These inflammatory signals can subsequently reach or influence the central nervous system, activate microglia, and amplify neuroinflammatory responses,^[45]^ ultimately altering brain function and contributing to depressive phenotypes.^[46,47]^ In addition to immune-inflammatory signaling, microbial metabolites constitute another major pathway of microbiota-gut-brain communication. Gut microorganisms can produce or modulate a wide range of neuroactive compounds, including neurotransmitters such as γ-aminobutyric acid, norepinephrine, dopamine, and serotonin, amino acids such as tryptophan, and microbial metabolites such as short-chain fatty acids. These molecules can interact with host metabolic and immune pathways after entering the portal circulation, while locally they may influence enteric neurons and vagal afferent signaling.^[48]^ These mechanistic observations provide biological context for the bibliometric finding that the gut-brain axis, inflammation, and neuroinflammation emerged as increasingly prominent research themes in the later stages of our thematic evolution analysis, reflecting a growing interest in the biological mechanisms underlying microbiota-depression interactions and antidepressant response.

Separately from the bibliometric findings, the clinical translational assessment indicated that the increasing convergence of gut microbiota-related research with antidepressant-related themes has begun to extend into human intervention studies. However, our assessment of the available clinical evidence suggests that this translational process remains at a relatively early stage. Among the 13 included clinical studies, 11 were randomized controlled trials, and 10 involved probiotic interventions, most of which were used as adjuncts to ongoing antidepressant treatment. Although some studies reported improvements in depressive symptoms, 2 studies did not find a significant benefit compared with placebo.^[37,38]^ The authors attributed these findings to their strict inclusion criteria, which excluded potential confounding factors such as unrecognized chronic inflammatory diseases, hormonal contraceptive use, or menstruation. Therefore, the included participants may have been subject to some degree of selection bias. In addition, the current clinical evidence is largely concentrated on probiotics, particularly Lactobacillus and Bifidobacterium, whereas mechanistic research suggests a broader range of potential microbiota-targeted strategies. Intervention durations were relatively short, ranging from 4 to 12 weeks, making it difficult to determine the long-term stability of microbiota changes and whether any clinical benefits could be sustained. However, some recent clinical trials have begun to incorporate microbiome, metabolomic, inflammatory, and neurobiological measures, including tryptophan metabolism, IL-6, and BDNF, suggesting that clinical research is gradually moving from symptom-based efficacy assessment toward a more mechanism-oriented approach. Future studies with larger sample sizes and longer follow-up are still needed, together with standardized microbiome profiling and mechanistic biomarkers, to further clarify whether microbiota changes mediate antidepressant effects or merely accompany the treatment process.

Several limitations of this study should be acknowledged. First, the bibliometric data were obtained exclusively from the WoSCC and Scopus. Although combining these 2 major databases broadened the literature coverage, relevant studies indexed only in other databases may have been missed. Moreover, only articles and reviews published in English were included, which may have introduced language and publication-type selection bias. Additionally, differences in metadata recording between WoSCC and Scopus, together with inconsistent terminology used for similar concepts across studies, may have introduced residual inconsistencies despite the standardization of duplicate records and relevant terms after database integration. Similarly, keyword- and citation-based indicators have inherent interpretive limitations. Author keywords are subjective and may not fully capture the core content of individual publications, potentially affecting thematic identification, while citation counts and citation bursts primarily reflect scholarly attention and can be influenced by publication age, database coverage, and temporal and disciplinary factors. Therefore, these indicators should be interpreted cautiously and should not be regarded as direct measures of methodological quality or clinical importance. Finally, the assessment of clinical studies was primarily intended to characterize the translational landscape of antidepressant-gut microbiota research rather than to systematically evaluate or quantitatively synthesize intervention efficacy. Accordingly, no formal methodological quality or risk-of-bias assessment was performed for the included clinical studies. Therefore, findings from the clinical component should be interpreted as reflecting the characteristics and translational trends of the current evidence base and should not be used to draw definitive conclusions regarding the efficacy of specific microbiota-targeted interventions or antidepressant treatment strategies. The absence of a formal quality assessment should also be considered when interpreting the strength of the clinical evidence.

## 5. Conclusions

This study maps the evolution of antidepressant-gut microbiota research from early gastrointestinal and psychological observations toward an increasingly integrated framework involving the microbiota-gut-brain axis, inflammation, microbial interventions, and antidepressant treatment. Although the field has expanded rapidly and clinical investigation is emerging, current translational evidence remains limited and heterogeneous. Future research should move beyond descriptive microbiome associations toward standardized, mechanism-informed, and adequately powered clinical studies to determine whether microbiota characteristics can serve as actionable therapeutic targets or predictors of antidepressant response.

## Author contributions

**Data curation:** Siliang Zeng, Wen Zheng, Dongfeng Wan.

**Investigation:** Siliang Zeng, Wen Zheng, Dongfeng Wan.

**Validation:** Siliang Zeng, Dongfeng Wan, Huili Chen.

**Conceptualization:** Huili Chen.

**Project administration:** Huili Chen.

**Supervision:** Huili Chen.

**Writing – original draft:** Siliang Zeng, Wen Zheng.

**Writing – review & editing:** Siliang Zeng, Dongfeng Wan, Huili Chen.

## References

[1] Grassi L Caruso R Riba MB; ESMO Guidelines Committee. Electronic address: clinicalguidelines@esmo.org. Anxiety and depression in adult cancer patients: ESMO Clinical Practice Guideline. ESMO Open. 2023;8:101155. 10.1016/j.esmoop.2023.101155 PMC10163167 37087199

[2] GBD 2023 Disease and Injury and Risk Factor Collaborators. Burden of 375 diseases and injuries, risk-attributable burden of 88 risk factors, and healthy life expectancy in 204 countries and territories, including 660 subnational locations, 1990-2023: a systematic analysis for the Global Burden of Disease Study 2023. Lancet. 2025;406:1873–922. 10.1016/S0140-6736(25)01637-X PMC12535840 41092926

[3] Bousman CA Stevenson JM Ramsey LB. Clinical Pharmacogenetics Implementation Consortium (CPIC) Guideline for CYP2D6, CYP2C19, CYP2B6, SLC6A4, and HTR2A Genotypes and Serotonin Reuptake Inhibitor Antidepressants. Clin Pharmacol Ther. 2023;114:51–68. 10.1002/cpt.2903 PMC10564324 37032427

[4] Harmer CJ Duman RS Cowen PJ. How do antidepressants work? New perspectives for refining future treatment approaches. Lancet Psychiatry. 2017;4:409–18. 10.1016/S2215-0366(17)30015-9 PMC5410405 28153641

[5] Klünemann M Andrejev S Blasche S. Bioaccumulation of therapeutic drugs by human gut bacteria. Nature. 2021;597:533–8. 10.1038/s41586-021-03891-8 PMC7614428 34497420

[6] Liu L Wang H Minichino A. Mapping the reciprocal interactions between antidepressants and the gut microbiome: novel targets for the personalisation and optimization of drug response. Mol Psychiatry. 2026;31:1992–2004. 10.1038/s41380-025-03345-x 41258378

[7] Zimmermann M Zimmermann-Kogadeeva M Wegmann R Goodman AL. Mapping human microbiome drug metabolism by gut bacteria and their genes. Nature. 2019;570:462–7. 10.1038/s41586-019-1291-3 PMC6597290 31158845

[8] Maier L Pruteanu M Kuhn M. Extensive impact of non-antibiotic drugs on human gut bacteria. Nature. 2018;555:623–8. 10.1038/nature25979 PMC6108420 29555994

[9] Vich Vila A Collij V Sanna S. Impact of commonly used drugs on the composition and metabolic function of the gut microbiota. Nat Commun. 2020;11:362. 10.1038/s41467-019-14177-z PMC6969170 31953381

[10] Savage N. The complex relationship between drugs and the microbiome. Nature. 2020;577:S10–1. 10.1038/d41586-020-00196-0 31996826

[11] Ellegaard O Wallin JA. The bibliometric analysis of scholarly production: How great is the impact? Scientometrics. 2015;105:1809–31. 10.1007/s11192-015-1645-z PMC4643120 26594073

[12] Ninkov A Frank JR Maggio LA. Bibliometrics: methods for studying academic publishing. Perspect Med Educ. 2022;11:173–6. 10.1007/s40037-021-00695-4 PMC9240160 34914027

[13] Thompson DF Walker CK. A descriptive and historical review of bibliometrics with applications to medical sciences. Pharmacotherapy. 2015;35:551–9. 10.1002/phar.1586 25940769

[14] Verma S Gustafsson A. Investigating the emerging COVID-19 research trends in the field of business and management: a bibliometric analysis approach. J Bus Res. 2020;118:253–61. 10.1016/j.jbusres.2020.06.057 PMC7330579 32834211

[15] Donthu N Kumar S Mukherjee D Pandey N Lim WM. How to conduct a bibliometric analysis: an overview and guidelines. J Bus Res. 2021;133:285–96.

[16] Jiang H Ling Z Zhang Y. Altered fecal microbiota composition in patients with major depressive disorder. Brain Behav Immun. 2015;48:186–94. 10.1016/j.bbi.2015.03.016 25882912

[17] Beurel E Toups M Nemeroff CB. The bidirectional relationship of depression and inflammation: double trouble. Neuron. 2020;107:234–56. 10.1016/j.neuron.2020.06.002 PMC7381373 32553197

[18] Valles-Colomer M Falony G Darzi Y. The neuroactive potential of the human gut microbiota in quality of life and depression. Nat Microbiol. 2019;4:623–32. 10.1038/s41564-018-0337-x 30718848

[19] Dinan TG Stanton C Cryan JF. Psychobiotics: a novel class of psychotropic. Biol Psychiatry. 2013;74:720–6. 10.1016/j.biopsych.2013.05.001 23759244

[20] Desbonnet L Garrett L Clarke G Bienenstock J Dinan TG. The probiotic Bifidobacteria infantis: an assessment of potential antidepressant properties in the rat. J Psychiatr Res. 2008;43:164–74. 10.1016/j.jpsychires.2008.03.009 18456279

[21] Burokas A Arboleya S Moloney RD. Targeting the microbiota-gut-brain axis: prebiotics have anxiolytic and antidepressant-like effects and reverse the impact of chronic stress in mice. Biol Psychiatry. 2017;82:472–87. 10.1016/j.biopsych.2016.12.031 28242013

[22] Desbonnet L Garrett L Clarke G Kiely B Cryan JF Dinan TG. Effects of the probiotic Bifidobacterium infantis in the maternal separation model of depression. Neuroscience. 2010;170:1179–88. 10.1016/j.neuroscience.2010.08.005 20696216

[23] Liu L Wang H Chen X Zhang Y Zhang H Xie P. Gut microbiota and its metabolites in depression: from pathogenesis to treatment. EBioMedicine. 2023;90:104527. 10.1016/j.ebiom.2023.104527 PMC10051028 36963238

[24] Liang S Wang T Hu X. Administration of Lactobacillus helveticus NS8 improves behavioral, cognitive, and biochemical aberrations caused by chronic restraint stress. Neuroscience. 2015;310:561–77. 10.1016/j.neuroscience.2015.09.033 26408987

[25] Wong ML Inserra A Lewis MD. Inflammasome signaling affects anxiety- and depressive-like behavior and gut microbiome composition. Mol Psychiatry. 2016;21:797–805. 10.1038/mp.2016.46 PMC4879188 27090302

[26] Li J Wang Y Li R. Adjunctive fecal microbiota transplantation for major depressive disorder: a randomized, double-blind, placebo-controlled trial. Cell Host Microbe. 2026;34:1367–82.e7. 10.1016/j.chom.2026.05.017 42309058

[27] Godzien J Kalaska B Rudzki L. Probiotic Lactobacillus plantarum 299v supplementation in patients with major depression in a double-blind, randomized, placebo-controlled trial: a metabolomics study. J Affect Disord. 2025;368:180–90. 10.1016/j.jad.2024.09.058 39271063

[28] Lee SM Dong TS Krause-Sorio B. The intestinal microbiota as a predictor for antidepressant treatment outcome in geriatric depression: a prospective pilot study. Int Psychogeriatr. 2022;34:33–45. 10.1017/S1041610221000120 33757609

[29] Sinha P Chatterjee P Kathiresan P. Efficacy of Adjunct PRObiotics as compared to the standard care in moderate unipolar depression among geriatric patients: a randomized double-blind placebo-controlled pilot multi-center trial (PRODG). J Am Geriatr Soc. 2026;74:2021–31. 10.1111/jgs.70530 PMC13418558 42304935

[30] Zhu X Wu S Zhou Y. The pharmacological actions of Danzhi-xiaoyao-San on depression involve lysophosphatidic acid and microbiota-gut-brain axis: novel insights from a systems pharmacology analysis of a double-blind, randomized, placebo-controlled clinical trial. J Biomol Struct Dyn. 2024;42:9309–24. 10.1080/07391102.2023.2251067 37632305

[31] Miyaoka T Kanayama M Wake R. Clostridium butyricum MIYAIRI 588 as adjunctive therapy for treatment-resistant major depressive disorder: a prospective open-label trial. Clin Neuropharmacol. 2018;41:151–5. 10.1097/WNF.0000000000000299 30234616

[32] Bambling M Edwards SC Hall S Vitetta L. A combination of probiotics and magnesium orotate attenuate depression in a small SSRI resistant cohort: an intestinal anti-inflammatory response is suggested. Inflammopharmacology. 2017;25:271–4. 10.1007/s10787-017-0311-x 28155119

[33] Reiter A Bengesser SA Hauschild AC. Interleukin-6 gene expression changes after a 4-week intake of a multispecies probiotic in major depressive disorder-preliminary results of the PROVIT study. Nutrients. 2020;12:2575. 10.3390/nu12092575 PMC7551871 32858844

[34] Nikolova VL Cleare AJ Young AH Stone JM. Acceptability, tolerability, and estimates of putative treatment effects of probiotics as adjunctive treatment in patients with depression: a randomized clinical trial. JAMA Psychiatry. 2023;80:842–7. 10.1001/jamapsychiatry.2023.1817 PMC10267847 37314797

[35] Kazemi A Noorbala AA Azam K Eskandari MH Djafarian K. Effect of probiotic and prebiotic vs placebo on psychological outcomes in patients with major depressive disorder: a randomized clinical trial. Clin Nutr. 2019;38:522–8. 10.1016/j.clnu.2018.04.010 29731182

[36] Tian P Chen Y Zhu H. Bifidobacterium breve CCFM1025 attenuates major depression disorder via regulating gut microbiome and tryptophan metabolism: a randomized clinical trial. Brain Behav Immun. 2022;100:233–41. 10.1016/j.bbi.2021.11.023 34875345

[37] Lin SK Kuo PH Hsu CY Chiu YH Chen CH. The effects of Lactobacillus plantarum PS128 in patients with major depressive disorder: an eight-week double-blind, placebo-controlled study. Asian J Psychiatr. 2024;101:104210. 10.1016/j.ajp.2024.104210 39232392

[38] Gawlik-Kotelnicka O Margulska A Płeska K Skowrońska A Strzelecki D. Metabolic status influences probiotic efficacy for depression-PRO-DEMET randomized clinical trial results. Nutrients. 2024;16:1389. 10.3390/nu16091389 PMC11085729 38732635

[39] Naseribafrouei A Hestad K Avershina E. Correlation between the human fecal microbiota and depression. Neurogastroenterol Motil. 2014;26:1155–62. 10.1111/nmo.12378 24888394

[40] Zheng P Zeng B Zhou C. Gut microbiome remodeling induces depressive-like behaviors through a pathway mediated by the host’s metabolism. Mol Psychiatry. 2016;21:786–96. 10.1038/mp.2016.44 27067014

[41] Aburto MR Cryan JF. Gastrointestinal and brain barriers: unlocking gates of communication across the microbiota-gut-brain axis. Nat Rev Gastroenterol Hepatol. 2024;21:222–47. 10.1038/s41575-023-00890-0 38355758

[42] Frieri M Stampfl H. Systemic lupus erythematosus and atherosclerosis: review of the literature. Autoimmun Rev. 2016;15:16–21. 10.1016/j.autrev.2015.08.007 26299985

[43] Pellegrini C Antonioli L Calderone V Colucci R Fornai M Blandizzi C. Microbiota-gut-brain axis in health and disease: is NLRP3 inflammasome at the crossroads of microbiota-gut-brain communications? Prog Neurobiol. 2020;191:101806. 10.1016/j.pneurobio.2020.101806 32473843

[44] Kelly JR Kennedy PJ Cryan JF Dinan TG Clarke G Hyland NP. Breaking down the barriers: the gut microbiome, intestinal permeability and stress-related psychiatric disorders. Front Cell Neurosci. 2015;9:392. 10.3389/fncel.2015.00392 PMC4604320 26528128

[45] Alexandrov PN Hill JM Zhao Y. Aluminum-induced generation of lipopolysaccharide (LPS) from the human gastrointestinal (GI)-tract microbiome-resident Bacteroides fragilis. J Inorg Biochem. 2020;203:110886. 10.1016/j.jinorgbio.2019.110886 PMC7409391 31707334

[46] Miller AH Haroon E Felger JC. Therapeutic implications of brain-immune interactions: treatment in translation. Neuropsychopharmacology. 2017;42:334–59. 10.1038/npp.2016.167 PMC5143492 27555382

[47] Miller AH Raison CL. The role of inflammation in depression: from evolutionary imperative to modern treatment target. Nat Rev Immunol. 2016;16:22–34. 10.1038/nri.2015.5 PMC5542678 26711676

[48] Morais LH Schreiber HL 4th Mazmanian SK. The gut microbiota-brain axis in behaviour and brain disorders. Nat Rev Microbiol. 2021;19:241–55. 10.1038/s41579-020-00460-0 33093662

